# Influence of Process Conditions During Aqueous and Direct Recycling of NMC811 Cathodes

**DOI:** 10.1002/cssc.202401803

**Published:** 2024-11-27

**Authors:** Felix Nagler, Leonhard Kolb, Nino Christian, Andreas Flegler, Michael Hofmann, Guinevere A. Giffin

**Affiliations:** ^1^ Fraunhofer R&D Center Electromobility Fraunhofer Institute for Silicate Research Neunerplatz 2 97082 Würzburg Germany; ^2^ Chair of Chemical Technology of Materials Synthesis Julius-Maximilians-University Würzburg Röntgenring 11 97070 Würzburg Germany

**Keywords:** Lithium-ion battery, direct recycling, aqueous processing, nickel-rich layered oxides, aluminum corrosion

## Abstract

This study investigated the impact of various process conditions on the aqueous, direct recycling of LiNi_0.8_Mn_0.1_Co_0.1_O_2_ (NMC811) cathodes. Three model systems were used. The first system assumes that the current collector delamination is performed in a dry environment without the use of water as a process medium. Consequently, the NMC811 is only exposed to water during classification, where no aluminum foil is present. The second model system assumes that the current collector delamination occurs in water. Therefore, the NMC811 is exposed to water in the presence of aluminum foil. Due to the pH increase caused by the Li^+^/H^+^ exchange reaction, the pH value surpasses the stability window of the aluminum‐oxide passivation layer (pH 4.5–8.5), resulting in the deposition of aluminum‐containing species on the NMC811 surface. The third model system is identical to the second, with the exception that H_3_PO_4_ is added. This causes the pH to decrease and prevents corrosion of the aluminum foil. The findings reveal that process conditions significantly affect the surface chemistry on NMC811, influencing electrochemical performance. Notably, aluminum‐containing species increase polarization. Heat treatment simulating regeneration led to cation mixing as surface species diffused into the NMC811 bulk structure, highlighting the need to control recycling process conditions.

## Graphical abstract

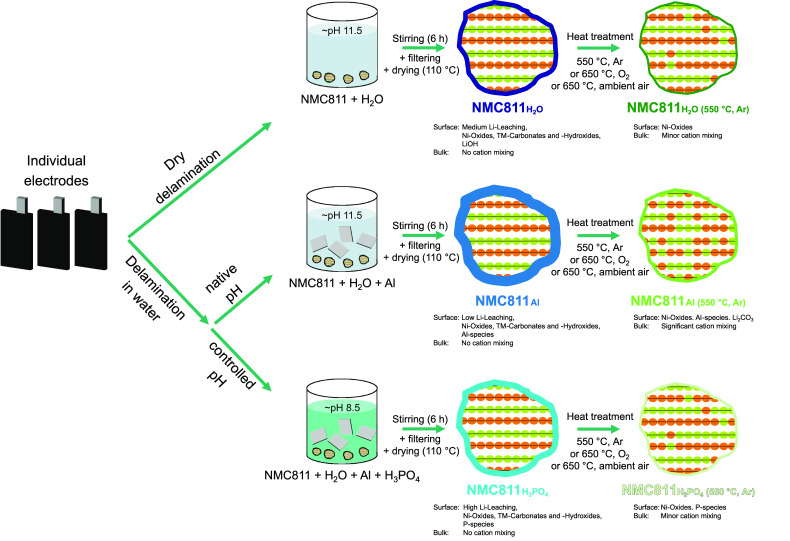

## Introduction

The increasing demand for lithium‐ion batteries (LIBs) and the limited availability of raw materials has made recycling of production scrap and end‐of‐life batteries crucial.[^1^, ^2^, ^3^, ^4^, ^5^] Direct recycling based on aqueous processes is likely to be a more sustainable alternative to conventional recycling methods.[^6^, ^7^, ^8^] Pyrometallurgy and hydrometallurgy often necessitate either energy or chemical intensive processes and yield only precursor materials. In contrast, direct recycling stands out for its ability to preserve the structure of crucial components, particularly that of the cathode active material.[^1^, ^9^] This would save a lot of energy as the synthesis of the cathode active material is one of the most energy consuming steps during LIB manufacturing.^[10]^

**Figure 1:**
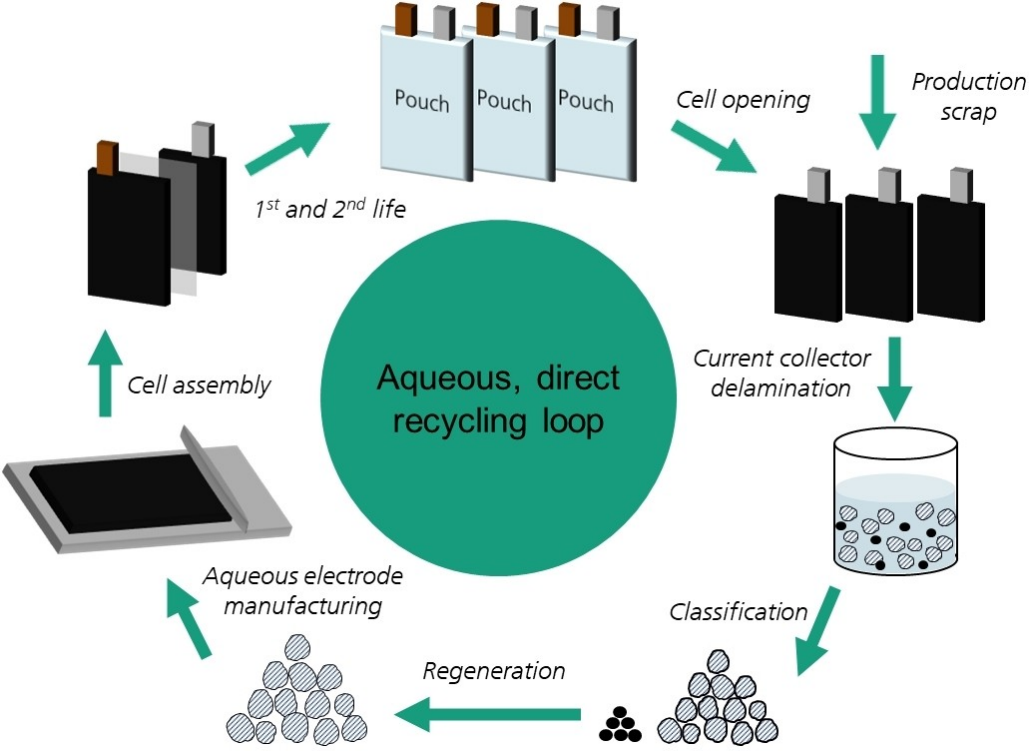
Schematic illustration of the aqueous, direct recycling process envisioned by Fraunhofer ISC.

In an aqueous, direct recycling process ‐ as envisioned at Fraunhofer ISC (schematically shown in Figure 1) ‐ the end of life (EOL) LIBs are opened individually after being sorted based on their cell chemistry. Then the different components of the cell – anode, cathode, separator – are separated from each other. The electrode composites of anode and cathode are delaminated from the current collectors. The electrode composites are classified to obtain clean fractions of the active materials and electrically conductive additives. Finally, the cathode active material needs to be regenerated, including a relithiation to compensate the loss of lithium during aging in the battery cell and recycling, before being used to produce new cathodes.

The exact process conditions of such a recycling process are crucial, especially for water sensitive materials like LiNi_0.8_Mn_0.1_Co_0._1O_2_ **(**NMC811). A Li^+^/H^+^ exchange takes place when the active material comes in contact with water, which leads to the formation of lithium and transition metal carbonates and hydroxides species on the NMC811 surface. These can lead to a reduction of cycling stability as they can trigger side reactions.[^11^, ^12^, ^13^, ^14^, ^15^, ^16^] During the recycling process, water contact of NMC811 is possible during current collector delamination and the classification of the electrode composite. The type and amount of surface species forming on the NMC811 surface might change depending on the exact process conditions. During the heat treatment, which usually is performed during the regeneration step, these surface species might further react or even diffuse into the bulk structure of the NMC811.

**Figure 2:**
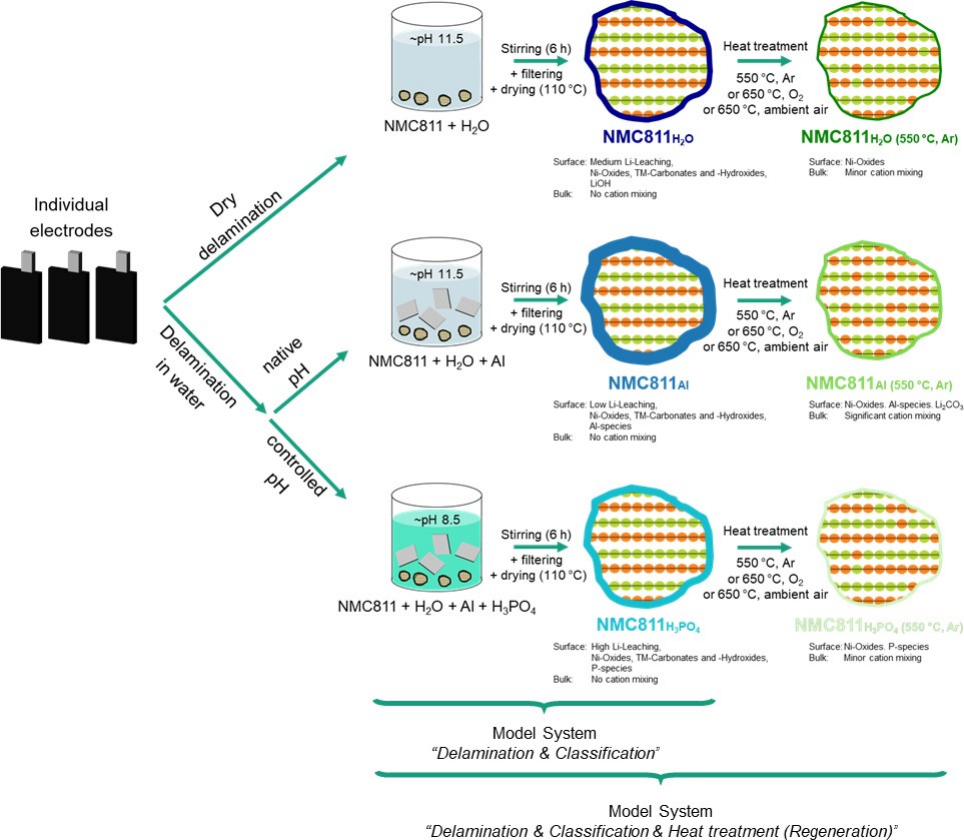
Illustration of the model systems for “Delamination & Classification” and “Delamination & Classification & Heat treatment (Regeneration)”. Both assume that the electrodes (anode and cathodes) can be separated after cell opening and that a clean process stream of cathodes can be generated.

In this study, the influence of the process parameters used during current collector delamination, classification and the heat treatment during regeneration of NMC811 cathodes were investigated. Pristine NMC811 material was used to effectively isolate the effects of the different process parameters applied during these steps. Given that in the short‐ to mid‐term, battery recycling, and particularly direct recycling, will focus on production scrap.[^17^, ^18^, ^19^] The results obtained with these model systems are expected to be directly applicable to production scrap samples. Moreover, the insights gained from these model systems will provide valuable information for the treatment of end‐of‐life materials. Characterization was conducted in two phases (Figure 2)) to gain a deeper understanding of the influence of process parameters across the different phases.

First, only the current collector delamination and classification were examined in different model systems, followed by characterization of the resultant active material to determine the condition of the NMC811 after these process steps. Second, the differently‐processed NMC811 were heat treated, as usually done during regeneration. Afterwards the NMC811 was characterized again. The characterization includes electrochemical measurements (cycling and electrochemical impedance measurements (EIS)) as well as scanning electron microscopy (SEM), thermogravimetry coupled with mass spectrometry measurements (TG‐MS), and X‐ray diffraction measurements (XRD). The process water was also analysed on the elemental composition via inductively coupled plasma optical emission spectrometry (ICP‐OES). The NMC811 was also characterized by X‐ray photoelectron spectroscopy (XPS) after the heat treatment.

## Results and Discussion

### Delamination & Classification

Initially, only the influence of different process conditions during current collector delamination and classification are investigated and the influence of the heat treatment during regeneration is excluded. Three different model systems were used for this purpose. For the first model system – immersion of the particles in water ‐ it was assumed that the delamination process takes place in a “dry” environment, i. e. without the use of water. For the second model system – immersion of the particles in water with aluminium ‐ a delamination process in water was assumed. The amount of aluminium foil was adjusted to represent cathodes with a mass loading of 15 mg/cm^2^. For the third model system – pH control with H_3_PO_4_ during immersion of the particles in water with aluminium ‐ it was assumed that the delamination process is performed in a pH‐controlled water bath so that no aluminium corrosion occurs (pH≤8.5). The NMC811 was added and the system was stirred for 6 h. Afterwards, the NMC811 was separated via filtering and dried (110 °C for 12 h). The various model systems are schematically shown in Figure 2b). The NMC811 particles were characterized electrochemically as well as via SEM, TG‐MS and XRD. The process water was analysed on the elemental composition via ICP‐OES. The active material samples of model systems one, two and three are called NMC811_H2O_, NMC811_Al_ and NMC811_H3PO4_, respectively.

The results of the ICP‐OES show (see Figure S1) that for the NMC811_H2O_ leaching of Li occurs, which can be identified in the process water (60 mg/L). For the NMC811_Al_, the amount of Li is reduced to 15 mg/L, but aluminium corrosion leads to the presence of Al species (30 mg/L) in the process water. It is also likely that Al‐containing species are present on the surface of the NMC811_Al_. As these poorly‐soluble surface species also contain lithium (see TG‐MS and XPS measurements), the lithium content in the process water is reduced in comparison to the NMC811_H2O_. In addition, it is possible that the formation of such surface species reduces the NMC811‐water interface for the NMC811_Al_, leading to less pronounced Li^+^/H^+^ exchange reaction. For the NMC811_H3PO4_, no Al‐species were detected in the process water, demonstrating that aluminium corrosion was successfully suppressed by the control of the pH with H_3_PO_4_. However, the addition of H_3_PO_4_ leads to a higher Li leaching (75 mg/L) due to the shift of the Li^+^/H^+^ exchange equilibrium. Additionally, a small amount of Ni (4 mg/L) is detected for the NMC811_H3PO4_, which indicates a small amount of transition metal leaching. This is explained by another leaching mechanism proposed by the Dahn group,^[20]^ which involves not only the leaching of lithium but also from transition metals. This leaching mechanism occurs at comparably low pH values. The upper pH limit for this leaching mechanism is dependent on the Ni concentration in the NMC structure. For LiNiO_2_, it is occurring at a pH of around 10. For NMC532, the pH is lower at ca. pH 7. For NMC811, this leaching mechanism may already take place to a small extent at a pH of 8.5.

**Figure 3:**
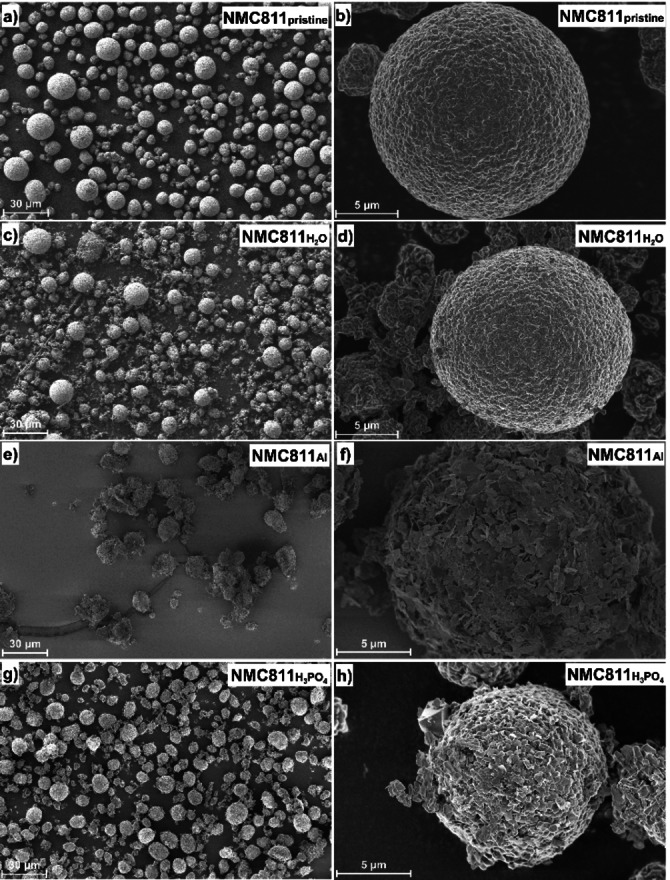
SEM images of a) and b) NMC811_pristine_, c) and d) NMC811_H2O_, e) and f) NMC811_Al_, g) and h) NMC811_H3PO4_.

SEM was used to examine the morphology of the different NMC811 surfaces (Figure 3). The NMC811_pristine_ secondary particles, which were not exposed to water at all, have a homogenous surface. In addition, the particles are almost all spherical. The images of NMC811_H2O_ show smaller secondary particles (see Figure 3c)), which means that particle cracking occurred. Smaller particles can also be detected on the surface of the NMC811_H2O_, which indicate the presence of surface species. The results for NMC811_H3PO4_ are similar to those of NMC811_H2O_, although the form of the surface species appears a bit different (see Figure 3g)‐h)). The morphology of the NMC811_Al_ is completely changed as compared to the NMC811_pristine_. The surface is covered with plate‐like particles (see Figure 3f)) and more agglomerates of secondary particles are observed (see Figure 3e)).

XRD measurements were performed to determine if the bulk of the differently‐processed NMC811 is changed (see Figure S2). The results suggest that no change as compared to the NMC811_pristine_ has occurred. All samples show nearly identical lattice parameters as well as identical ratios of the reflection intensities of (102), (006) and (101). According to Dahn et al.,^[21]^ the ratio is an indication for Li^+^ and Ni^2+^ cation mixing. A higher the ratio R ((I(102)+I(006))/I(101)) of these reflections indicates a higher cation mixing as the intensity of (101) reflection (at around 36.5 °) decreases if Ni^2+^ occupies Li sites in the layered structure.^[22]^ Cation mixing seems not to be present extensively. This conclusion is further supported by the refinement of the Ni occupancy on the transition metal sites and lithium sites. The refinement suggests that for the NMC811_pristine_, 3.1 % of the nickel atoms are located on lithium sites. This is only slightly higher for the processed NMC811, where around 4 % of the nickel atoms are located on the lithium sites (see Table S3).

**Figure 4:**
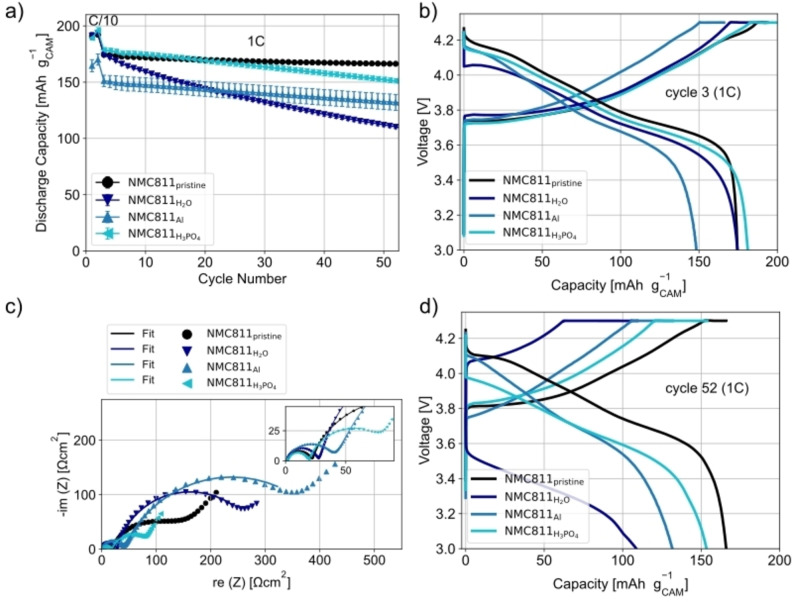
Electrochemical characterization of the NMC811 from the various model recycling processes. NMP/PVDF‐based cathodes were produced to “freeze” surface after processing. Furthermore, the cathodes were tested in half cells against a lithium metal counter electrode to ensure the relithiation of the NMC811. a) Mean specific discharge capacity of three cells in voltage window of 3.0–4.3 V at 25 °C. Error bars are the standard deviation of the three cells tested. b) Voltage profile of one representative cell in the 3^rd^ cycle (1^st^ cycle at 1 C). c) Nyquist plot and fits of EIS measurements performed at 3.6 V from 10 mHz to 1 MHz. d) Voltage profile of one representative cell in the 52^nd^ cycle (50^th^ cycle at 1 C).

For the electrochemical characterization, cathodes with the NMC811 were produced and tested vs. a lithium metal counter electrode. Conventional cathode processing was performed, i. e., using NMP and a PVDF‐binder, to “freeze” the surface of the NMC811 as much as possible and to avoid exposing the NMC811 to water again. The cycling data of the differently‐processed NMC811 shows differences in the initial discharge capacity (DC). In the 3^rd^ cycle (1^st^ cycle at 1 C), the NMC811_pristine_ has a DC of 174 mAh/g_CAM_ (CAM=cathode active material). The cells with NMC811_H2O_ and NMC811_H3PO4_ show similar DCs with 174 mAh/g_CAM_ and 178 mAh/g_CAM_ respectively. Even though lithium is leached from the CAM structure out during the model recycling processes and surface species are formed, the lithium metal anode is able to compensate this loss of lithium. This explains the similar DC that is achieved for the NMC811_H2O_ and NMC811_H3PO4_ as compared to NMC811_pristine_. This is not the case for the NMC811_Al_. In the 3^rd^ cycle, a DC of only 151 mAh/g_CAM_ is achieved. This leads to the conclusion that the aluminium corrosion during the model recycling process and the resultant surface species initially induce additional polarizations. The voltage profiles of cycle 3 (Figure 4b)) show that the polarization for the cells with NMC811_Al_ is higher during discharge than that of the other cells especially at lower states of charge (SOC). The EIS data at 3.6 V in Figure 4c) shows that NMC811_Al_ has the highest charge transfer resistance R_CT_ (semi‐circle at low frequencies[^14^, ^16^]) with 394.3 Ωcm^2^. All fit values are presented in Table S1. The NMC811_pristine_, NMC811_H2O_ and NMC811_H3PO4_ show R_CT_ of 140.1, 268.8 and 78.8 Ωcm^2^ respectively. The smaller R_CT_ of the NMC811_H3PO4_ compared to the NMC811_pristine_ could originate from the presence of phosphate surface species due to the addition of H_3_PO_4_, which could can facilitate the lithium‐ion transport. Similar effects were observed by Yao et al.^[23]^ who coated LiNi_1/3_Co_1/3_Mn_1/3_O_2_ by adding H_3_PO_4_.

The semi‐circle at high frequencies is attributed to the surface film resistance, which has contributions from both the solid electrolyte interphase on the lithium metal counter electrode and the surface layer formed on the cathode particles during the model recycling processes. This assignment is consistent with results previously published investigating for the effect of aqueous electrode processing on the cathode surface chemistry.[^14^, ^16^] R_f_ is higher for the NMC811_Al_ (40.1 Ωcm^2^) than all other samples, which have R_f_ values of around 20 Ωcm^2^. It is likely that the aluminium corrosion which occurs during the model recycling process leads to the formation of aluminium‐based surface species and the increased resistance value. The resistance corresponding mainly to the impedance of the liquid electrolyte, R_IR_ (high frequency intercept), is similar for all samples with values around 1.5 Ωcm^2^.

During cycling all NMC811 exposed to the model recycling process conditions show capacity fading. In contrast, the NMC811_pristine_ shows a very stable cycling with a DC of 166 mAh/g_CAM_ in the 52^nd^ cycle (50^th^ cycle at 1 C) and a capacity retention of 95 %.

The NMC811_H2O_ shows the highest capacity fading with a DC of 110 mAh/g_CAM_ and a capacity retention of 63 %. In contrast, the cells with NMC811_Al_, which had the lowest initial DC, have a capacity retention of 87 % (DC of 132 mAh/g_CAM_). However, the relatively good cyclability of the NMC811_Al_ could result from the lower amount of cycled Li due to the much higher initial polarization and the lower DCs. As a result, the degradation of the lithium metal counter electrode is less, which could lead to a better cycling performance. It is also possible that the surface species, induced by the model recycling processes, lead an improved cycling stability by limiting side reactions with the electrolyte at the NMC811 surface. The NMC811_H3PO4_ shows the best cycling performance. After 50 cycles at 1 C, the cells show a DC of 151 mAh/g_CAM_ and a capacity retention of 85 %. The improved cyclability of the NMC811_H3PO4_ compared to the NMC811_H2O_ can be attributed to the presence of phosphoric surface species, which act as a surface coating. In a recent study by these authors, H_3_PO_4_ was used to coat NMC811 and the resulting phosphate surface species led to a much better cyclability as they at least partially suppress side reactions with the electrolyte at the NMC811 surface.^[12]^ As can be seen in the voltage profile of the 52^nd^ cycle (50^th^ cycle at 1 C), the polarizations of the NMC811_H3PO4_ are much smaller than those observed for the NMC811_H2O._ All relevant DCs as well as the capacity retentions are summarized in Table S2.

The composition and amount of the species present on the surface of the differently‐processed NMC811 were investigated with TG‐MS measurements. The results (see Figure S3) show that the NMC811_Al_ clearly has the highest mass loss of 3.8 % up to 700 °C. In contrast, NMC811_H2O_ and NMC811_H3PO4_ show only weight losses of 1.1 % and 1.4 %, respectively. This indicates that the amount of surface species created during processing is significantly higher for the NMC811_Al_. The weight loss above 700 °C originates mainly from the decomposition of the NMC811 itself.^[24]^ For temperatures below 700 °C the MS data in Figure S3c) shows that mainly water is responsible for the weight loss of the NMC811_Al_. From 150–400 °C a broad peak of H_2_O is observed. According to literature, this peak could originate from Al‐containing species like hydroxides (e. g. LiAl_2_(OH)_7_) or carbonates (e. g. Li_2_Al_4_CO_3_(OH)_12_). These are known to have associated water, which is released starting at around 150 °C.[^25^, ^26^] In addition to H_2_O, there is also a small release of oxygen starting at about 300 °C. This could have several origins. One possibility would be that NiOOH was formed during processing, which is known to decompose to NiO and O_2_ around 300 °C.^[27]^ Another possibility would be that the Li^+^/H^+^ exchange reaction leads to the formation of Li_x_NiO_2_ with x <0.5. Such a delithiated phase would transform to LiNiO_2_ and LiNi_2_O_4_ with the release of oxygen in the temperature range of 300 °C, according to literature.[^28^, ^29^] The MS data of NMC811_Al_ shows an additional CO_2_ peak at around 600 °C. This could be explained by the decomposition of Al‐containing carbonates itself like e. g., Li_2_Al_4_CO_3_(OH)_12_. According to Drewien et al.,^[25]^ Li_2_Al_4_CO_3_(OH)_12_ decomposes to Al_2_O_3_, LiAlO_2_ and LiAl_5_O_8_ with the release of CO_2_ in this temperature range. The O_2_ peak in the same temperature range indicates an ongoing reduction reaction. Species such as LiNi_2_O_4_ ‐ if it is present ‐ or nickel oxides, where the nickel is in an oxidation state > +II, could be reduced. In a previous publication, it was observed that Mn‐oxides tend to be reduced in a temperature range above 600 °C.^[30]^ Besides aluminium species, it is also possible that a part of the mass loss observed for the NMC811_Al_ is associated with the decomposition of species like NiCO_3_ and Ni(OH)_2_. According to Sicklinger et al.,^[24]^ these species decompose between 180–400 °C and release H_2_O and CO_2_. The TG‐MS spectra of NMC811_Al_ is quite complex and the signals could be attributed to a variety of different species, as described in the text above. This suggests that the presence of Al leads to a complex surface chemistry, which must be addressed during the subsequent recycling processes.

In the case of the NMC811_H2O_ and NMC811_H3PO4_, these Ni‐species might be mainly responsible for the weight loss since no Al‐containing species are expected for these samples. The NMC811_H2O_ shows a further weight loss between 400–600 °C, whereas the NMC811_H3PO4_ produces no signal in this temperature range. The associated surface species decomposing in this range are CoCO_3_, MnCO_3_ as well as LiOH.[^11^, ^24^] The presence of these species might explain the poorer cycling performance (see Figure 4a)). In particular, LiOH is known to be responsible for a decrease in cycling performance.^[31]^

In summary, the different conditions of the three model processes lead to very different electrochemical cycling data for the NMC811. As the bulk structure of all samples is nearly identical to the NMC811_pristine_, the differences in the surface chemistry is responsible for this result. Although NMC811_H2O_ has the lowest mass loss below 600 °C and thus the lowest amount of surface species, the moieties like LiOH lead to poorer cycling stability. Al corrosion during the model recycling process for NMC811_Al_ leads to the formation of Al‐containing surface species, which induce high polarizations in the electrochemical cells and the comparably low DCs. The best electrochemical performance is shown by cells with NMC811_H3PO4_. The surface species occurring in this case have the least impact on the capacity and cycling stability, resulting in an electrochemical performance that is closest to the NMC811_pristine_.

### Delamination & Classification & Heat treatment (Regeneration)

A heat treatment step is commonly applied during or prior to the regeneration phase in direct recycling processes.^[6]^ To evaluate the impact of this step on the active material, the NMC811 samples produced using the three model recycling processes described above were subjected to heat treatment for 1 hour at 550 °C in an argon atmosphere. Additionally, heat treatments were also performed in oxygen and ambient atmospheres at 650 °C for 1 hour each. These variations were included to reflect the wide range of regeneration procedures currently being developed, which employ different atmospheric conditions during heat treatment, as reported in the literature.^[6]^

As discussed above, the surface chemistry depends on the different process conditions. During the heat treatment, these surface species could decompose, transform or even diffuse into the bulk of the NMC811. It is clear that the exact process conditions of the regeneration (e. g. solvents and Li salts during relithiation, etc.) could vary and thus, it is possible, that – at least partially – the removal (via washing) or a transformation of some surface species occurs. Nonetheless, for the purposes of this study to obtain an initial understanding of the influence of the surface species from previous process steps, specific regeneration conditions were not taken into account. The heat treatment is used as a model for the regeneration step.

**Figure 5:**
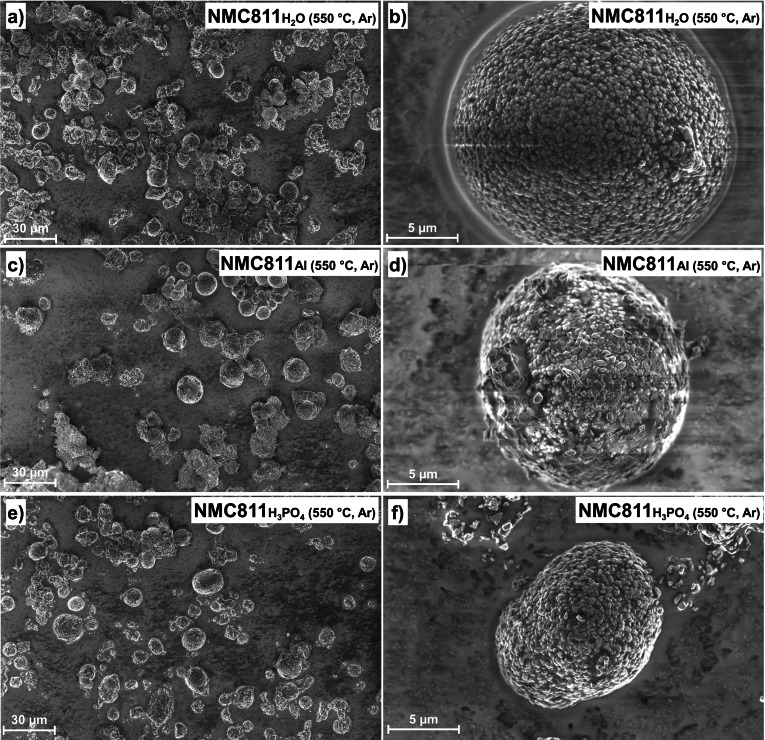
SEM images of a)+b) NMC811_H2O (550 °C, Ar)_, e)+f) NMC811_Al (550 °C, Ar)_, g)+h) NMC811_H3PO4 (550 °C, Ar)_.

The NMC811 surface was visualized in SEM images (see Figure 5). The morphology of the NMC811_H2O (550 °C, Ar)_ and NMC811_H3PO4 (550 °C, Ar)_ are similar to that of the samples without heat treatment (Figure 3d) and h), respectively). For both samples, relatively smooth surfaces are observed. In contrast, the images reveal that the morphology of the NMC811_Al (550 °C, Ar)_ is changed after the heat treatment. Some of the plate‐like surface species, which were covering the complete surface before the heat treatment (see NMC811_Al_ in Figure 3f)), have disappeared. In Figure S4, SEM images of NMC811 particles, which were treated in oxygen (650 °C, 1 h) are shown. The surface morphology does not significantly differ when the heat‐treatment atmosphere is changed to oxygen. The NMC811_H2O (650°C, O2)_ and NMC811_H3PO4 (650°C, O2)_ show relatively smooth surfaces, comparable to the samples without heat treatment. For the NMC811_Al (650°C, O2)_, a portion of the plate‐like surface species, which were observed for the NMC811_Al_, disappeared, but less than in the case of NMC811_Al (550°C, Ar)_.

XRD measurements were performed to determine if heat treatment affects the bulk structure of the NMC811. The results indicate that indeed the bulk structure is changed after the heat treatment and depends on the surface chemistry before the heat treatment (see Figure S2). An increased cation ion mixing is expected for all heat‐treated NMC811 as the ratio (I_[102]_ +I_[006]_)/I_[101]_ increases. The highest value of 0.77 is observed for the NMC811_Al (550 °C, Ar)_, followed by NMC811_H3PO4 (550 °C, Ar)_ with 0.62 and NMC_H2O (550 °C, Ar)_ with 0.57. As a reference, the NMC811_pristine_ has a value of 0.52 for this intensity ratio. Refinement of the nickel site occupancy also reveals an increase in cation mixing. About 10.4 % of Ni atoms are located on Li sites for NMC811_Al (550 C, Ar),_ 5.9 % for the NMC811_H2O (550 C, Ar)_ and 6.6 % for NMC811_H3PO4 (550 C, Ar)._ In the reference NMC811_pristine_, only 3.0 % of the Ni atoms are located on Li sites. The reason for the increased cation mixing within the NMC811 structures might be the diffusion of components of the surface species into the bulk of the NMC811 during the heat treatment. Since for NMC811_Al_ has the highest amount of surface species (see TG‐MS measurements in Figure S3), this could explain why NMC811_Al (550 °C, Ar)_ shows the highest cation mixing. Finally, a slight increase of the lattice parameters is observed for all heat‐treated NMC811. The XRD measurement of the NMC811 heat treated in an oxygen atmosphere show similar trends for the intensity ratio indicating cation mixing and for the lattice parameters (see Table S3). The NMC811_Al (650°C, O2)_ has the highest (I[102] +I[006])/I[101] ratio of 0.77, followed by the NMC811_H3PO4 (650°C, O2)_ with 0.62 and the NMC811_H2O (650°C, O2)_ with 0.61. In addition, the refinement of the XRD data to yield the nickel site occupancy shows that the cation mixing depends on the combination of process conditions and heat treatment parameters. In an O_2_ atmosphere, NMC811_Al (650°C, O2)_ has the highest percentage of Ni in the Li sites with 8.1 %, followed by NMC811_H3PO4 (650°C, O2)_ with 6.3 % and NMC811_H2O (650°C, O2)_ with 4.9 %. The biggest difference between a heat treatment in an argon and oxygen atmosphere is evident for the NMC811_Al (650°C, O2)_ sample, where the Ni occupancy of lithium sites decreases by roughly 2.5 %. This result, in combination with that from the SEM images in Figure S4, suggests that amount of the surface species of NMC811_Al (550°C, Ar)_ is lower, but the removal of these species seems to lead to more cation mixing. In the case of NMC811_Al (650°C, O2),_ the amount of surface species visible in the SEM image is higher (more plate‐like structures), but the cation mixing in the bulk structure is lower. These results suggest that the influence of the heat treatment atmosphere is complex in that the nature and stability of the surface species in a given atmosphere subsequently affect the bulk cathode structure.

To examine the surface chemistry of the heat‐treated NMC811, TG‐MS as well as XPS measurements were performed for the samples heated in argon atmosphere. The TG‐MS measurements in Figure S5 show that only very small mass losses occur for all samples up to 700 °C (0.2 % for NMC811_H2O (550 °C, Ar)_; 0.4 % for NMC811_Al (550 °C, Ar)_ and NMC811_H3PO4 (550 °C, Ar)_). All samples show a mass loss at temperatures above 700 °C, which can be attributed to the decomposition of the NMC811 structure itself.^[24]^ The MS measurements show a O_2_ signal, which is consist with the structural decomposition. The NMC811_Al (550 °C, Ar)_ shows an additional CO_2_ peak at around 800 °C, which can be attributed to the decomposition of Li_2_CO_3_. No other decomposition of surface species can be observed, which means that any other remaining surface species are stable up to 1100 °C under the conditions tested.

**Figure 6:**
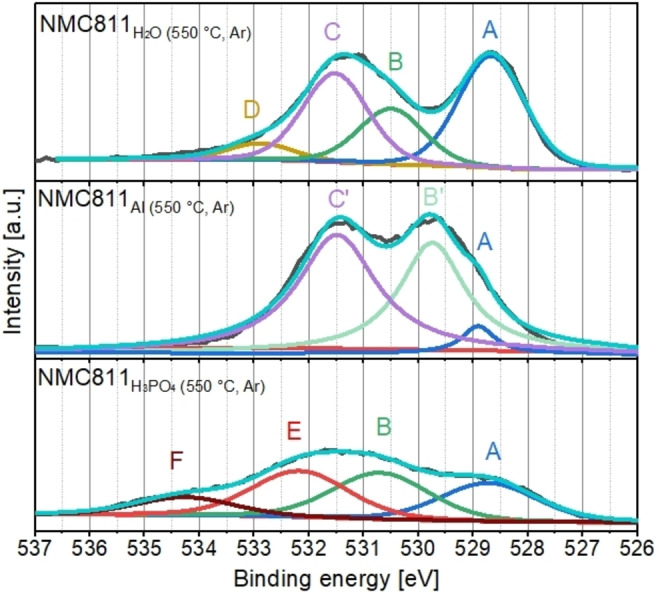
O1s spectra of XPS measurement. Peak A corresponds to NMC811, peaks B and B’ to NiO, peak C to Ni_2_O_3_, peak C’ to Ni_2_O_3_, Li_2_CO_3_, LiAlO_2_, LiAl_5_O_8_, and Al_2_O_3,_ peak D to absorbates, peak E and F to P_2_O_5_ species.

The XPS of the O1s region of the heat‐treated samples show several spectral features, which can be separated into six different peaks (see Figure 6). Peak A at 529.0 eV is present for all samples and can be attributed to oxygen in the NMC811 structure itself. This implies that none of the samples is covered by surface species to a degree that the NMC811 cannot be detected. Peak B at 530.5 eV, can be attributed to NiO. For the NMC811_Al (550 °C, Ar)_, this peak is slightly shifted to 529.8 eV (and thus labelled as B’). According to Khawaja et al.,^[32]^ NiO has two peaks in the O1s spectrum at 528.7 eV and 530.6 eV. Fleisch et al,^[33]^ who also observed two peaks for NiO in the O1s spectrum, but at slightly other binding energies, proposed that the peak splitting originates in the presence of so‐called “defect oxygen”. This leads to a mixture of NiO and other nickel oxides (NiO_x_, x >2). Therefore, it is possible that NMC811_Al (550 °C, Ar)_ has a slightly different NiO_x_ composition, leading to a small shift of the peak. Peak C at 531.5 eV for NMC811_H2O (550 °C, Ar)_ can be assigned to Ni_2_O_3_.^[34]^ All other possible species typically present at this binding energy (e. g., transition metal carbonates and hydroxides) should not be thermal stable and are decomposed during heat treatment. Only Li_2_CO_3_ could be possible, since it is thermally stable up to 800 °C. However, there is no CO_2_ signal present in the TG‐MS measurement of the NMC811_H2O (550 °C, Ar)_ (Figure S5c)).

For NMC811_Al (550 °C, Ar)_, there is also a peak at 531.5 eV (peak C’). However, in this case other species can be contribute to this signal aside from Ni_2_O_3_. Since a CO_2_ signal is present in the TG‐MS measurement of the NMC811_Al (550 °C, Ar)_ (Figure S5c)), Li_2_CO_3_ is likely to be present on the particle surface. According to literature, Al‐containing species like LiAlO_2_,[^35^, ^36^] LiAl_5_O_8_ ^[37]^ and Al_2_O_3_,^[38]^ which are known to be possible decomposition products of Li_2_Al_4_CO_3_(OH)_12_, could also contribute to peak C’. The Al2p spectrum (Figure S6) clearly shows the presence of Al‐containing surface species for NMC811_Al (550 °C, Ar)_. These species are very likely to be present on the surface after the heat treatment. Peak D for NMC811_H2O (550 °C, Ar)_ at 533.0 eV can be attributed to absorbates.^[39]^ Peak E and F at 532.2 eV and 534.3 eV for NMC811_H3PO4 (550 °C, Ar)_ can attributed to P_2_O_5_ containing species. This peak splitting occurs since the oxygen in P_2_O_5_ is present in a P‐O and P‐O‐P configuration, which results in slightly different binding energies of the oxygen.^[40]^ P_2_O_5_‐containing species are plausible, since H_3_PO_4_ was used to control the pH during processing of NMC811_H3PO4 (550 °C, Ar)_. The likely surface species for the heat‐treated NMC811 are summarized in Table 1 and are derived by combining the TG‐MS and XPS measurement results.

**Table 1:** Summary of the likely surface species of heat‐treated NMC811 resulting from the analysis of the TG‐MS and XPS measurements.

Sample	Surface species
NMC811H_2_O (550 °C, Ar)	NiO, Ni_2_O_3_
NMC811Al (550 °C, Ar)	NiO, Ni_2_O_3_, LiAlO_2_, LiAl_5_O_8_, Al_2_O_3_, Li_2_CO_3_
NMC811H_3_PO_4_ (550 °C, Ar)	NiO, P_2_O_5_

**Figure 7:**
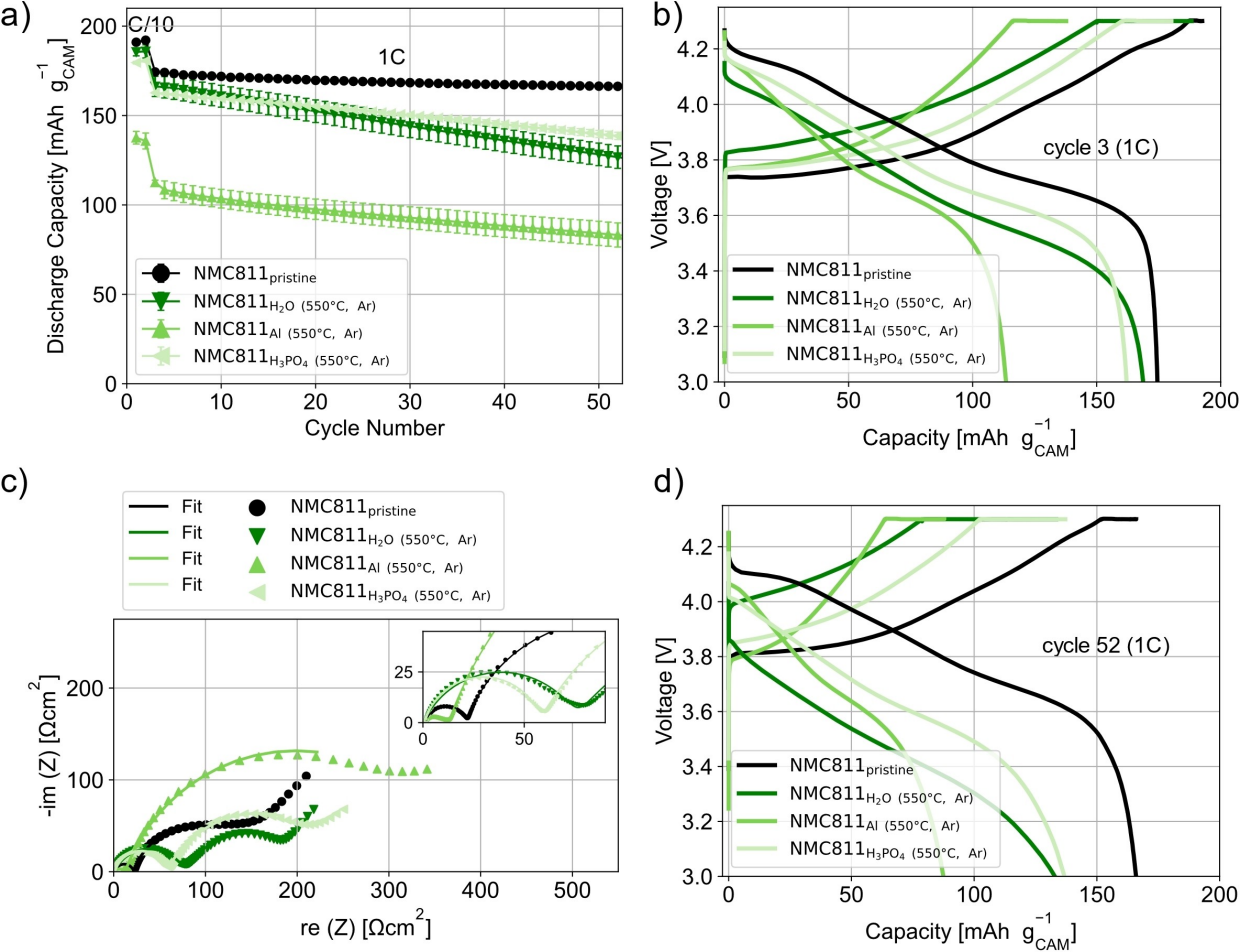
Electrochemical characterization of differently‐processed NMC811 after a heat treatment. NMP/PVDF‐based cathodes were produced to “freeze” surface after processing. The cathodes were tested in half cells against a lithium metal counter electrode to ensure the relithiation of the NMC811. a) Mean specific discharge capacity of three cells vs cycles in voltage window of 3.0‐4.3 V at 25 °C. Error bars are the standard deviation of the three cells. b) Voltage profile of one representative cell in the 3^rd^ cycle (1^st^ cycle at 1 C). c) Nyquist plot and fits of EIS measurements performed at 3.6 V from 10 mHz to 1 MHz. d) Voltage profile of one representative cell in the 52^nd^ cycle (50^th^ cycle at 1 C).

As described above, NMP/PVDF‐based cathodes were prepared for the electrochemical characterization of the NMC811 materials. At the beginning of cycling, all heat‐treated NMC811 samples have lower DC than the NMC811_pristine_ and also lower DC than the respective samples before the heat treatment. The lower DCs can be attributed to cation mixing which occurs during the heat treatment. Cation mixing reduces the Li‐ion mobility and results in the increased polarisation observable in the voltage profiles in Figure 7b). The significantly lower DCs of both the NMC811_Al (550 °C, Ar)_ (see Figure 7) and NMC811_Al (650°C, O2)_ (see Figure S7) compared to the heat‐treated samples with different process conditions can be explained by higher cation mixing occurring with these two materials. While the heat‐treatment parameters have only a small influence on the DCs for the other two process conditions, there is a more significant difference between the DCs of NMC811_Al (550 °C, Ar)_ and NMC811_Al (650°C, O2)_. In this case, the DCs of NMC811_Al (650°C, O2)_ is higher, which may suggest that the effect of cation mixing is more detrimental than the presence of Al‐containing surface species.

The impedance measurements at 3.6 V of the heat‐treated samples in Figure 7c) show that the NMC811_Al (550 °C, Ar)_ has the highest R_CT_ value of 372.4 Ωcm^2^ (all fit values in Table S4). The NMC811_H2O (550 °C, Ar)_ and NMC811_H3PO4 (550 °C, Ar)_ show R_CT_ values of 150.8 and 200.2 Ωcm^2^, respectively. The surface species present on the surface of the NMC811_Al (550 °C, Ar)_ induce the high charge transfer resistance. These high values along with the more significant cation mixing are consistent with the high polarization observed in the voltage profiles.

Surprisingly, the resistance associated with the surface film R_f_ is the lowest for the NMC811_Al (550 °C, Ar)_ with a value of 12.3 Ωcm^2^. The differences in the R_f_ are assumed to come primarily from variations in the cathode surface layers. Large differences in the SEI resistance are not expected during the early stages of cycling (i. e., directly after formation). NMC811_Al (550 °C, Ar)_ is the only heat‐treated sample in an argon atmosphere where Li_2_CO_3_ is likely present. Li_2_CO_3_ is known to be a comparably good lithium ion conductor,^[41]^ which could explain the smaller R_f_ value as the lithium ion migration through the cathode surface layer might be enhanced. The R_f_ values are higher for the other heat‐treated samples, where Li_2_CO_3_ is not present. The R_f_ values of the NMC811_H2O (550 °C, Ar)_ and NMC811_H3PO4 (550 °C, Ar)_ are 75.4 Ωcm^2^ and 56.9 Ωcm^2^, respectively. The R_IR_ is quite similar for all sample and between 0.3‐1.5 Ωcm^2^.

During cycling, capacity fading is observable for all heat‐treated samples. In contrast, as noted above, the capacity retention of the NMC811_pristine_ is 95 %. The NMC811_H3PO4 (550 °C, Ar)_ shows the best cycling performance of the heat‐treated samples, with a capacity retention of 86 %. The NMC811_H2O (550 °C, Ar)_ shows a higher capacity fading, but both the DC and capacity retention are better than that of the material (NMC811_H2O_) before heat treatment (DC in 52^nd^ cycle of 127 mAh/g_CAM_ and capacity retention of 77 % for NMC811_H2O (550 °C, Ar)_ vs DC in 52^nd^ cycle of 110 mAh/g_CAM,_ capacity retention 63 % for NMC811_H2O_). This might be explained by the removal of species like LiOH, which are known to be detrimental for cycling stability.^[31]^ The highest capacity fading for the heat‐treated samples is observed for the NMC811_Al (550 °C, Ar)_. The DC in the 52^nd^ cycle is only 83 mAh/g_CAM_, which is equal to a capacity retention of 73 %. All relevant DC and capacity retention values are summarized in Table S2.

In summary the heat treatment leads to the increase of the cation mixing in the bulk NMC811 structure. This is most pronounced for the NMC811_Al (550 °C, Ar)_ and results in significant polarization, leading to lower DCs. For the NMC811_H2O (550 °C, Ar)_ and NMC811_H3PO4 (550 °C, Ar)_, this effect is less pronounced, most likely because less surface species are present before the heat treatment. For the NMC811_H2O (550 °C, Ar)_, a slightly better cycling stability is observed after the heat treatment, indicating that the removal or decomposition of some surface species leads to a better cyclability. In general, the results reveal that it is crucial to understand the amount and type of species present on the NMC811 surface before performing a heat treatment. Based on the type of surface species, the heat‐treatment can lead to an improvement of the cycling performance (NMC811_H2O_) or to a significant increase in polarization (NMC811_Al_). To close the performance gap to NMC811_pristine_, complete removal of the surface species might be necessary after water exposure of the NMC811.

## Conclusions

The study reveals the importance of controlling the process conditions during aqueous and direct recycling. The use of different model recycling processes combined with characterization of the resulting surface species shows that the amount and type of surface species varies depending on the process conditions. The NMC811_H3PO4_ shows the best electrochemical performance. The use of H_3_PO_4_ during processing successfully suppresses the aluminum current collector corrosion and simultaneously forms P‐containing surface species, which protects the NMC811. In contrast, without pH control, as with NMC811_Al_, aluminum current collector corrosion occurs, ultimately resulting in increased polarization during cycling. This polarization is associated with the formation of Al‐based surface species. The worst electrochemical performance is shown by the NMC811_H2O_, where the NMC811 is only processed in water. In this case, it is the type (most notably LiOH) rather than the amount of surface species which proves to be particularly detrimental for the performance. The surface chemistry is also critical in subsequent process steps, such as a heat treatment, as these species can lead to cation mixing. It is likely that components of the surface species diffuse into the bulk of the NMC811 structure. This cation ion mixing leads to increased polarizations and hence lower DC during cycling. This was most notable for the NMC811_Al (550 °C, Ar)_, which had the most surface species present before the heat treatment. The nature of the surface species can also affect the cation mixing. The other two samples (NMC811_H2O (550 °C, Ar)_ and NMC811_H3PO4 (550 °C, Ar)_) show a similar electrochemical performance after the heat treatment. Since capacity fading is observed for all samples, the removal of the surface species generated during the recycling processes might be necessary before a heat treatment, as would be the case in regeneration, to close the gap in electrochemical performance to the pristine active material.

## Experimental Section

### Model recycling processes

NMC811 (Gelon LiB Group) was subjected to three different model recycling processes. The first model system used only deionized water (DI‐water). The NMC811‐DI‐water ratio was adjusted to be 1 : 50 (5 g NMC811 : 250 g DI‐water). The second model system was identical to the first, but with the addition of aluminum foil. The amount of aluminum foil represents the amount equal to a cathode foil with a mass loading of 15 mg/cm^2^. Hence, for 5 g of NMC811, 333.33 cm^2^ of aluminum foil was added to the system (25 pieces of (2.6×2.6) cm^2^). The third model system was identical to model system two, but H_3_PO_4_ (12.5 wt % H_3_PO_4_ in H_2_O, Sigma Aldrich Chemie GmbH) was added to control the pH to a range between 7.5 and 8.5. In all model systems, the mixtures were constantly stirred on a magnetic stirrer with 300 rounds per minute for 6 h. For the third system, H_3_PO_4_ was added as following: 620 mg after 1 min, 200 mg after 15 min, 150 mg after 120 min and 100 mg after 300 min. The pH was constantly checked with pH‐meter (PCE‐Instruments Deutschland GmbH, PCE‐228). After 6 h, the NMC811 was filtered with a glass microfiber filter (Sartorius Lab Instruments GmbH & Co. KG, MG 1387/1). Subsequently, the NMC811 was pre‐dried over night at 110 °C and then dried further in the glovebox oven at 1 mbar and 110 °C for 12 h.

For thermal treatments the samples were placed in zirconium crucibles and put in ovens under specific atmospheres. For the heat treatment under argon a sintering furnace (CAF, MUT Advances Heating GmbH) was used, for the heat treatment under oxygen atmosphere a tube furnace (SR 11/100/500, Carbolite Gero GmbH & Co. KG) and for the heat treatment under ambient atmosphere a muffle furnace (P330, Nabertherm GmbH). The samples were heated with a heating rate of 5 K/min to 550 °C (argon atmosphere) and 650 °C (oxygen and ambient atmosphere) and held here for 1 h. After cooling down the samples got transferred to the glovebox with a drying procedure at 1 mbar and 110 °C for 12 h. A heat treatment at 650 °C was also attempted under argon atmosphere. However, the material could not be processed into electrodes afterward because the cathode composite delaminated from the aluminum current collector following doctor blading and drying. This might be attributed to the earlier onset of NMC811 decomposition in an argon environment. According to TG‐MS measurements conducted with a heating rate of 10 K/min, decomposition begins around 700 °C under argon, compared to around 750 °C under oxygen (see Figures S3 and S8). Given that TG‐MS is a dynamic measurement with a higher heating rate, the actual onset temperature of the decomposition during a static heat treatment may be slightly lower. Consequently, the decomposition threshold could have been reached during the static heat treatment at 650 °C under argon. Therefore, the temperature of the heat treatment under argon was reduced to 550 °C.

### Electrode processing

To prepare cathodes, a slurry consisting of 92 wt % NMC811, 4 wt % polyvinylidene difluoride (PVDF, Solvay GmbH, Solef 5130) as binder and 4 wt % Super C65 (C‐NERGY™, Imerys S.A.) as conductive carbon was used. The PVDF was dissolved in NMP (Sigma Aldrich Chemie GmbH) overnight before adding the NMC811 and the Super C65. The slurry was mixed for 2 h with a Speedmixer (DAC 400.1 Vac‐P, Hauschild GmbH & Co. KG). The amount of NMP was adjusted to 120 wt % compared to the powders. The wet gap size during the doctor blade coating was adjusted to 200 μm. After coating the slurry on aluminum foil, the solvent was evaporated in the fume hood overnight and then in a vacuum oven for 2 h at 80 °C. The cathodes were calendered to a porosity of about 50 %. Disc electrodes with a diameter of 16 mm were punched and dried under vacuum for 12 h at 110 °C.

### Half‐cell tests against lithium metal counter electrode

The cathodes were tested in half cells against lithium metal (thickness 300 μm, Sigma‐Aldrich Chemie GmbH) on a copper substrate as counter electrode, a microporous separator (Celgard LLC, Celgard 2500) and 200 μl of 1 mol/L LiPF_6_ in EC/DMC 1 : 1 wt%/wt % (LP30, BASF SE) with the addition of 5 wt % of fluoroethylene carbonate (FEC, Sigma Aldrich Chemie GmbH) as electrolyte. A ceramic knife was used to scrape the surface of the lithium metal to remove the oxide layer. Subsequently, the lithium metal was rolled onto dendritic copper (SE‐Cu R360, Schlenk Metallfolien GmbH & Co. KG). The cells were assembled in an argon‐filled glovebox (GS‐Glovebox, O_2_ <1.0 ppm, H_2_O <0.1 ppm). Three cells of each cathode batch were prepared. Aluminum and nickel‐copper tabs (Targray Battery & Energy Storage) were used to contact cathode and anode, respectively. Cyclization tests were performed on an electrochemical workstation (Maccor, Series 4000, Maccor Inc.) in the voltage range of 3.0 V to 4.3 V at 25 °C (IPP260, Memmert GmbH + Co. KG). The cells were charged with a constant‐current constant‐voltage procedure (CCCV) and discharged with a constant‐current procedure (CC). The CV‐step was terminated at a current of C/20.

EIS measurements were performed on a VMP300 galvanostat/potentiostat (BioLogic GmbH). Before measuring the impedance spectra, the cells were cycled three times with C/10 for cell formation. A CV step of 3 h at 3.6 V was applied before the impedance measurements to ensure that the cells were at steady‐state. The impedance spectra were obtained by the perturbation of the cells with an AC voltage (amplitude: 5 mV) over the frequency range of 10 mHz to 1 MHz.

### Characterization methods

ICP‐OES analysis of the filtrate from the water‐exposure experiments was performed on a Vista‐Pro (Varian Inc.) spectrometer. The detection limits for lithium, aluminum and nickel were 0.006 mg/L, 0.02 mg/L and 0.03 mg/L, respectively. pH measurements were performed with a pH meter (PCE‐Instruments Deutschland GmbH, PCE‐228) using an electrode designed for the high alkaline range (WTW, SenTix®H) at a temperature of 25±2 °C.

The XRD of the samples were performed with a SmartLab diffractometer (Rigaku Corporation) in the range of 2θ=10–80 °, at a scan rate of 0.5°/min. The Rietveld refinements were performed with the SmartLab Studio II software using the full parameter approach. Here the lattice parameters, the crystallite size as well as the nickel occupancy on lithium and nickel sites were refined.

Coupled TG‐MS measurements (NETZSCH STA 449 C Jupiter® coupled with NETZSCH QMS 403 C Aëolos®) were performed between 50–1100 °C in argon with a heating rate of 10 K/min.

XPS spectra were recorded using a Surface Science Instruments S‐Probe X‐ray photoelectron spectrometer with an Al‐Kα monochromatic X‐ray source. Before the measurement, the powders were embedded into indium foil. The spectra were calibrated to the C1s peak at 284.6 eV.

SEM measurements were performed with a ZEISS Supra 25 (Carl Zeiss Microscopy GmbH).

## Conflict of Interests

The authors declare no conflict of interest.

1

## Supporting information

As a service to our authors and readers, this journal provides supporting information supplied by the authors. Such materials are peer reviewed and may be re‐organized for online delivery, but are not copy‐edited or typeset. Technical support issues arising from supporting information (other than missing files) should be addressed to the authors.

Supporting Information

## Data Availability

The data that support the findings of this study are available from the corresponding author upon reasonable request.

## References

[1] L. Gaines , Q. Dai , J. T. Vaughey , S. Gillard , Recycling 2021, 6, 31.

[2] J. B. Goodenough , K.-S. Park , J. Am. Chem. Soc. 2013, 135, 1167. 23294028 10.1021/ja3091438

[3] M. Li , J. Lu , Z. Chen , K. Amine , Adv. Mater. 2018, 30, e1800561 . 10.1002/adma.201800561 29904941

[4] T. Kim , W. Song , D.-Y. Son , L. K. Ono , Y. Qi , J. Mater. Chem. A 2019, 7, 2942.

[5] M. Göttlinger , P. Daubinger , W. Stracke , S. Hartmann , G. A. Giffin , Electrochim. Acta 2022, 419, 140354.

[6] H. Ji , J. Wang , J. Ma , H.-M. Cheng , G. Zhou , Chem. Soc. Rev. 2023, 52, 8194. 37886791 10.1039/d3cs00254c

[7] Z. Zhuang , J. Li , H. Ji , Z. Piao , X. Wu , G. Ji , S. Liu , J. Ma , Di Tang , N. Zheng , J. Wang , G. Zhou , Adv. Mater. 2024, 36, e2313144. 38441371 10.1002/adma.202313144

[8] J. Wang , H. Ji , J. Li , Z. Liang , W. Chen , Y. Zhu , G. Ji , R. Shi , G. Zhou , H.-M. Cheng , Nat Sustain 2024, 7, 1283.

[9] G. Wei , Y. Liu , B. Jiao , N. Chang , M. Wu , G. Liu , X. Lin , X. Weng , J. Chen , L. Zhang , C. Zhu , G. Wang , P. Xu , J. Di , Q. Li , iScience 2023, 26, 107676. 37680490 10.1016/j.isci.2023.107676 PMC10480636

[10] Q. Dai , J. C. Kelly , L. Gaines , M. Wang , Batteries 2019, 5, 48.

[11] F. Nagler , N. Christian , P. Daubinger , A. Flegler , M. Hofmann , G. A. Giffin , J. Power Sources 2023, 24, 100131.

[12] F. Nagler , N. Christian , A. Gronbach , F. Stahl , P. Daubinger , A. Flegler , M. Hofmann , G. A. Giffin , ChemElectroChem 2024, 11, A170.

[13] M. Hofmann , M. Kapuschinski , U. Guntow , G. A. Giffin , J. Electrochem. Soc. 2020, 167, 140512.

[14] M. Hofmann , M. Kapuschinski , U. Guntow , G. A. Giffin , J. Electrochem. Soc. 2020, 167, 140535.

[15] M. Hofmann , F. Nagler , U. Guntow , G. Sextl , G. A. Giffin , J. Electrochem. Soc. 2021, 168, 60511.

[16] M. Hofmann , F. Nagler , M. Kapuschinski , U. Guntow , G. A. Giffin , ChemSusChem 2020, 13, 5962. 32969581 10.1002/cssc.202001907 PMC7756629

[17] A. Wolf , F. Nagler , P. Daubinger , C. Neef , K. Mandel , A. Flegler , G. A. Giffin , Energy Environ. Sci. 2024, 41, 304.

[18] A. Breiter, M. Linder, T. Schuldt, G. Siccardo, N. Vekic **2023**, https://www.mckinsey.com/industries/automotive-and-assembly/our-insights/battery-recycling-takes-the-drivers-seat. Accessed 13 September 2024.

[19] M. Ahuis , A. Aluzoun , M. Keppeler , S. Melzig , A. Kwade , J. Power Sources 2024, 593, 233995.

[20] I. Hamam , N. Zhang , A. Liu , M. B. Johnson , J. R. Dahn , J. Electrochem. Soc. 2020, 167, 130521.

[21] J. Dahn , Solid State Ionics 1990, 44, 87.

[22] E.-J. Lee , Z. Chen , H.-J. Noh , S. C. Nam , S. Kang , D. H. Kim , K. Amine , Y.-K. Sun , Nano Lett. 2014, 14, 4873. 24960550 10.1021/nl5022859

[23] Y. Yao , H. Liu , G. Li , H. Peng , K. Chen , Electrochim. Acta 2013, 113, 340.

[24] J. Sicklinger , M. Metzger , H. Beyer , D. Pritzl , H. A. Gasteiger , J. Electrochem. Soc. 2019, 166, A2322–A2335.

[25] C. A. Drewien , D. R. Tallant , M. O. Eatough , J. Mater. Sci. 1996, 31, 4321.

[26] M. Nayak , T. R. N. Kutty , V. Jayaraman , G. Periaswamy , J. Mater. Chem. 1997, 7, 2131.

[27] M. S. Hamdan , Riyanto , M. R. Othman , Int. J. Electrochem. Sci. 2013, 8, 4747.

[28] J. Dahn , E. Fuller , M. Obrovac , U. Vonsacken , Solid State Ionics 1994, 69, 265.

[29] J. Morales , C. Pérez-Vicente , J. L. Tirado , J. Therm. Anal. 1992, 38, 295.

[30] M. Rumpel , F. Nagler , L. Appold , W. Stracke , A. Flegler , O. Clemens , G. Sextl , Mater Adv 2022, 52, 279.

[31] N. V. Faenza , L. Bruce , Z. W. Lebens-Higgins , I. Plitz , N. Pereira , L. F. J. Piper , G. G. Amatucci , J. Electrochem. Soc. 2017, 164, A3727–A3741.

[32] E. E. Khawaja , M. A. Salim , M. A. Khan , F. F. Al-Adel , G. D. Khattak , Z. Hussain , J. Non-Cryst. Solids 1989, 110, 33.

[33] T. Fleisch , N. Winograd , W. N. Delgass , Surf. Sci. 1978, 78, 141.

[34] L. Salvati , L. E. Makovsky , J. M. Stencel , F. R. Brown , D. M. Hercules , J. Phys. Chem. 1981, 85, 3700.

[35] W. Tang , Z. Chen , F. Xiong , F. Chen , C. Huang , Q. Gao , T. Wang , Z. Yang , W. Zhang , J. Power Sources 2019, 412, 246.

[36] A. Tesfay Reda , D. Zhang , X. Xu , J. Environ. Chem. Eng. 2022, 10, 107842.

[37] B. Qin , Y. Wu , R. Qiu , J. Ruan , ACS Sustainable Chem. Eng. 2023, 11, 1386.

[38] J. A. Rotole , P. M. A. Sherwood , Surf. Sci. Spectra 1998, 5, 18.

[39] B. P. Payne , M. C. Biesinger , N. S. McIntyre , J. Electron Spectrosc. Relat. Phenom. 2009, 175, 55.

[40] B. V. R. Chowdari , K. L. Tan , W. T. Chia , R. Gopalakrishnan , J. Non-Cryst. Solids 1990, 119, 95.

[41] J. Mizusaki , H. Tagawa , K. Saito , K. Uchida , M. Tezuka , Solid State Ionics 1992, 53–56, 791.

