# Supplementary material for: Influence of Process Conditions During Aqueous and Direct Recycling of NMC811 Cathodes
Source: ChemSusChem. 2024 Nov 27;18(7):e202401803. doi: 10.1002/cssc.202401803 (PMC11960599; doi:10.1002/cssc.202401803)
Supplement: Supplementary file 1 — Supporting Information [file CSSC-18-e202401803-s001.pdf]

ChemSusChem

Supporting Information

Influence of Process Conditions During Aqueous and Direct Recycling of NMC811 Cathodes

Felix Nagler, Leonhard Kolb, Nino Christian, Andreas Flegler, Michael Hofmann, and Guinevere A. Giffin*

Supporting Information

Influence of Process Conditions during Aqueous and Direct Recycling

Felix Nagler[a], Leonhard Kolb[a], Nino Christian[a], Andreas Flegler[a], Michael Hofmann[a], Guinevere A. Giffin*1[a, b]

[a] Felix Nagler, Leonhard Kolb, Nino Christian, Dr. Andreas Flegler, Dr. Michael Hofmann, Dr. Guinevere A. Giffin
Fraunhofer R&D Center Electromobility
Fraunhofer Institute for Silicate Research
Neunerplatz 2, 97082 Würzburg, Germany
E-mail: guinevere.giffin@isc.fraunhofer.de

[b] Dr. Guinevere A. Giffin
Chair of Chemical Technology of Materials Synthesis
Julius-Maximilians-University Würzburg
Röntgenring 11, 97070 Würzburg, Germany

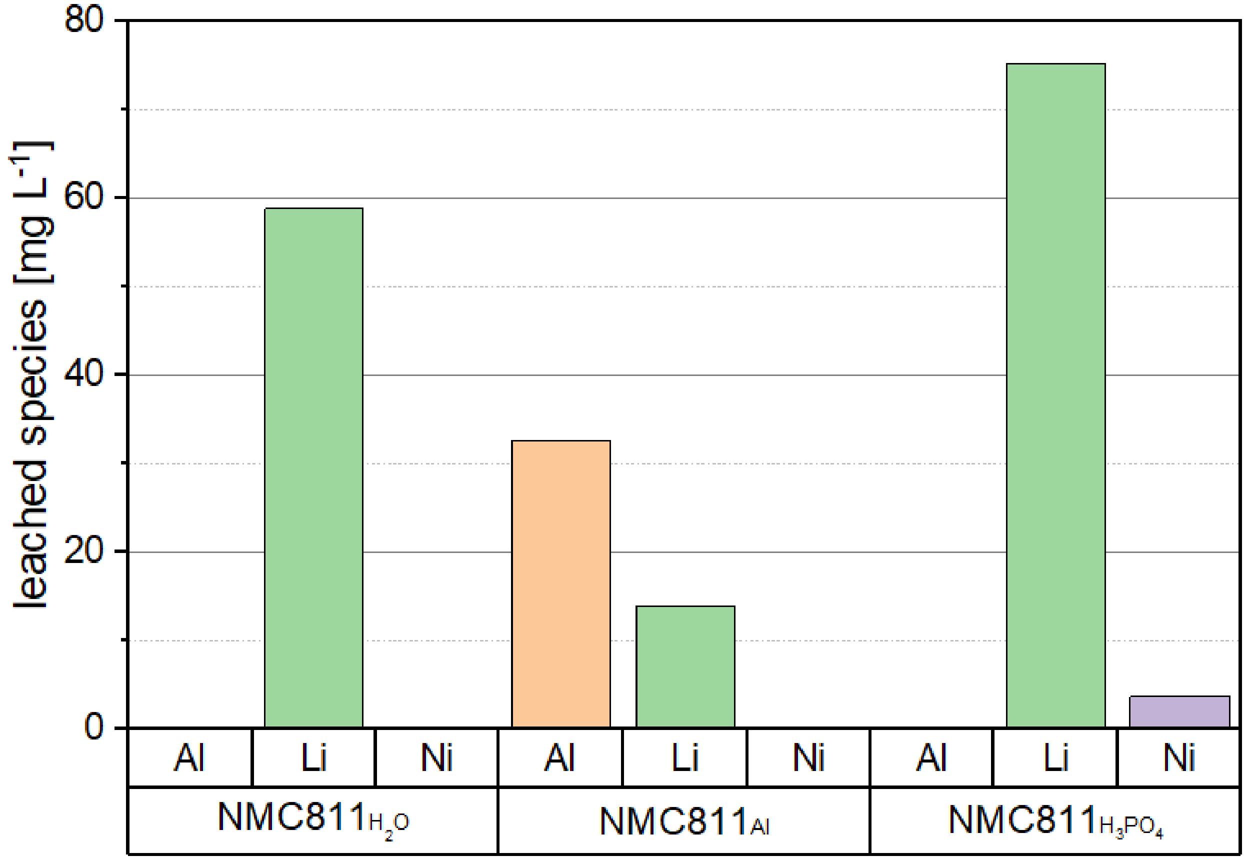

Figure S1: ICP-OES measurements of process water of NMC811$_{H2O}$, NMC811$_{Al}$, NMC811$_{H3PO4}$.

a)

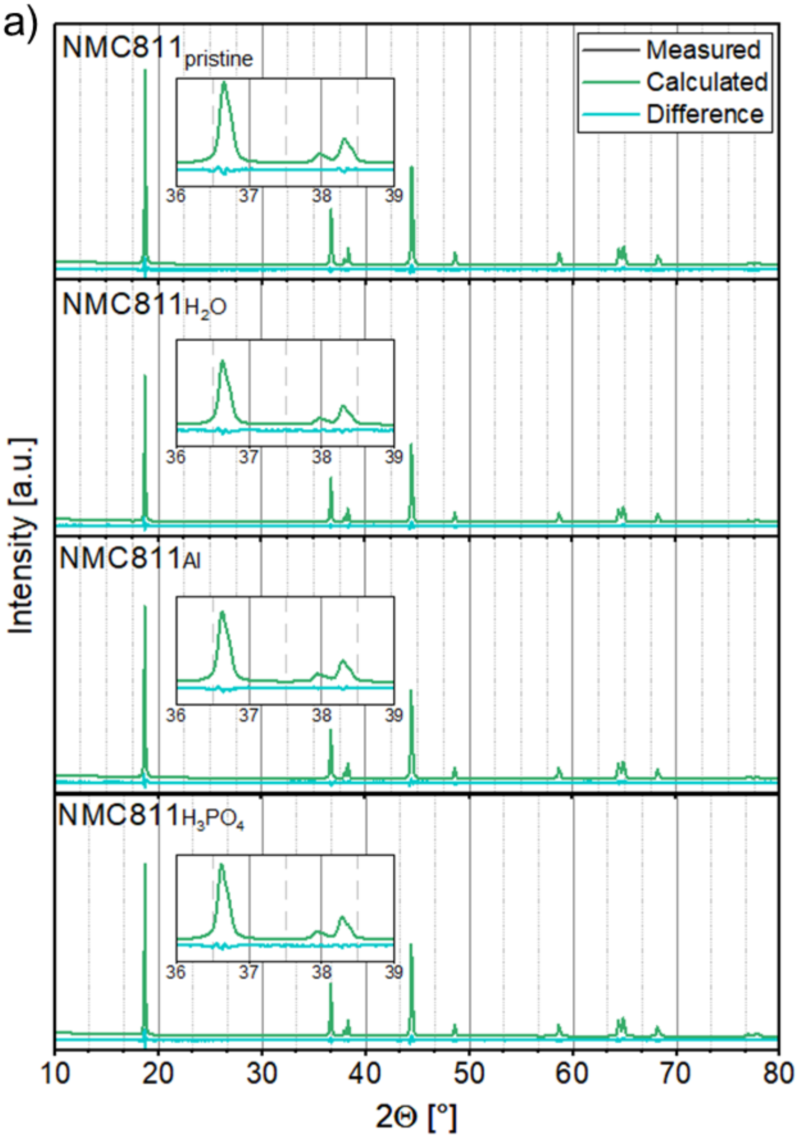

Measured
Calculated
Difference
NMC811$_{pristine}$
NMC811$_{H_2O}$
NMC811$_{Al}$
NMC811$_{H_3PO_4}$
36
37
38
39
Intensity [a.u.]
10
20
30
40
50
60
70
80
2Θ [°]

b)

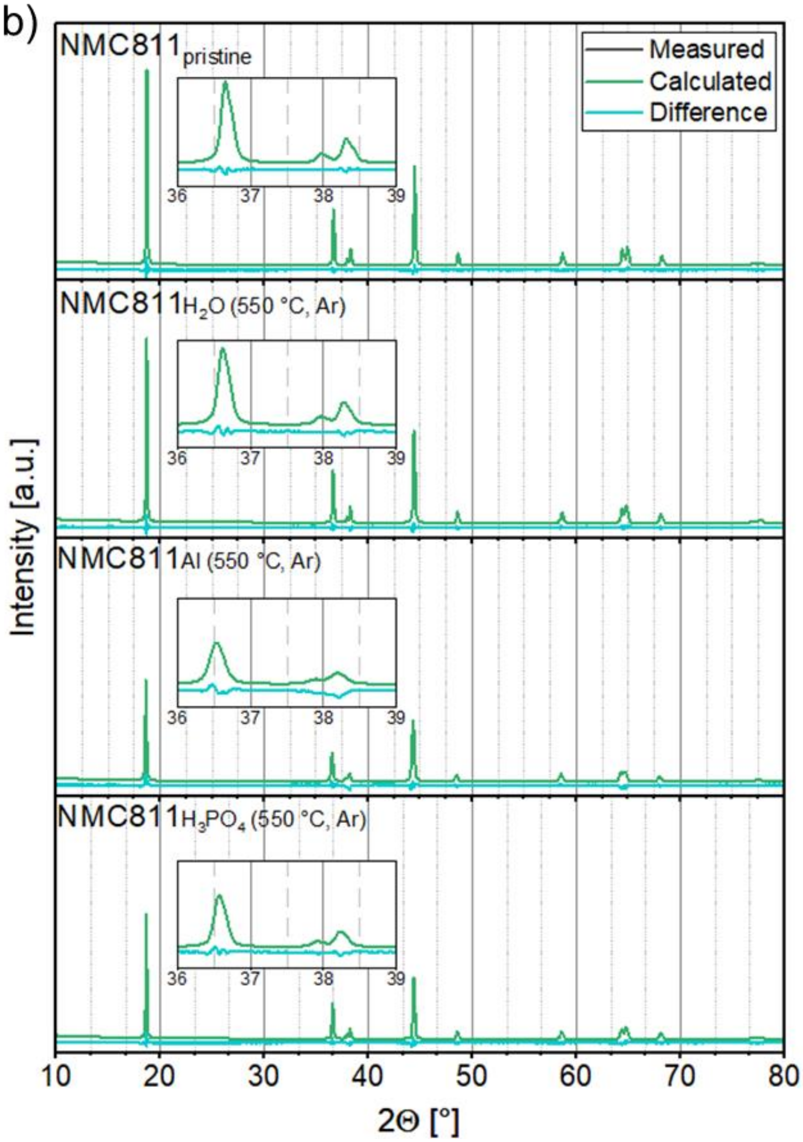

NMC811pristine
Measured
Calculated
Difference
NMC811H2O (550 °C, Ar)
NMC811Al (550 °C, Ar)
NMC811H3PO4 (550 °C, Ar)
36
37
38
39
Intensity [a.u.]
10
20
30
40
50
60
70
80
2Θ [°]

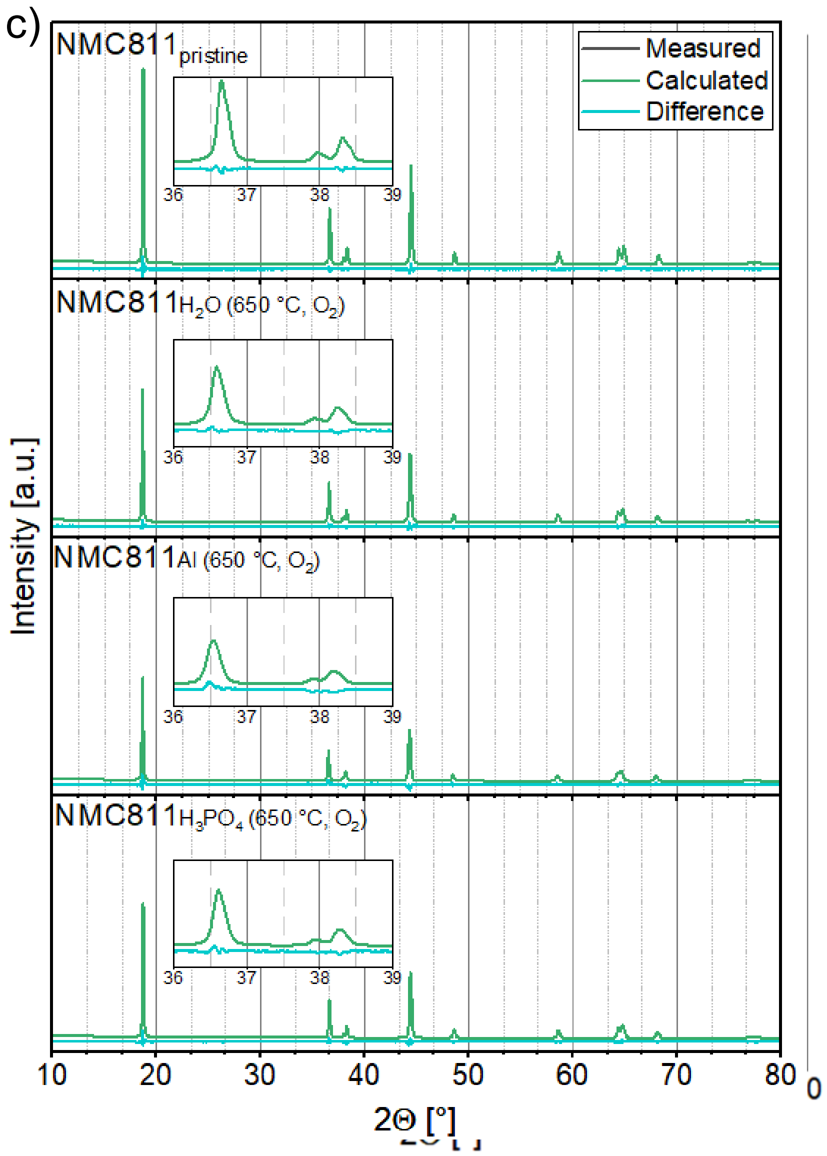

Figure S2: XRD measurements of differently-processed NMC811, a) without heat treatment, b) with heat treatment in argon atmosphere (550 °C, 1 h), c) with heat treatment in oxygen atmosphere (650 °C, 1 h). h) and c) with heat treatment in oxygen atmosphere (650 °C, 1 h). The structural data such as the lattice parameters were extracted by performing Rietveld refinements with the SmartLAB Studio II software.

Table S1: Resistance values from the fits of EIS measurements at 3.6 V.

	R_{IR}	R_{SEI}	R_{CT}
$NMC811_{pristine}$	1.5	20.0	140.1
$NMC811_{H_2O}$	1.2	26.2	268.8
$NMC811_{Al}$	1.0	40.1	394.3
$NMC811_{H_3PO_4}$	1.4	17.4	78.8

Table S2: DCs of the 3rd and 52nd cycle, which is the 1st and 50th cycle at 1C, and the capacity retention.

	DC (3rd cycle) in mAh/g_{CAM}	DC (52nd cycle) in mAh/g_{CAM}	Capacity retention
$NMC811_{pristine}$	174	166	95%
$NMC811_{H2O}$	174	110	63%
$NMC811_{Al}$	151	132	87%
$NMC811_{H3PO4}$	178	151	85%
$NMC811_{H2O}$ (550 °C, Ar)	166	127	77%
$NMC811_{Al}$ (550 °C, Ar)	113	83	73%
$NMC811_{H3PO4}$ (550 °C, Ar)	162	129	86%
$NMC811_{H2O}$ (650 °C, O2)	174	127	73%
$NMC811_{Al}$ (650 °C, O2)	122	92	75%
$NMC811_{H3PO4}$ (650 °C, O2)	172	148	86%
$NMC811_{H2O}$ (650 °C, ambient air)	176	136	77%
$NMC811_{Al}$ (650 °C, ambient air)	139	124	89%
$NMC811_{H3PO4}$ (650 °C, ambient air)	172	142	83%

Table S3: Structural data extracted from Rietveld refinements of XRD measurements in Figure S2.

	$R = \frac{I_{(102)}+I_{(006)}}{I_{(101)}}$	Percentage of Ni on Li sites	a in Å	c in Å
$NMC811_{pristine}$	0.52	3.0	2.872	14.208
$NMC811_{H_2O}$	0.54	4.1	2.873	14.207
$NMC811_{Al}$	0.54	3.7	2.873	14.208
$NMC811_{H_3PO_4}$	0.55	4.1	2.872	14.209
$NMC811_{H_2O}$ (550 °C, Ar)	0.57	5.5	2.875	14.213
$NMC811_{Al}$ (550 °C, Ar)	0.77	10.4	2.878	14.226
$NMC811_{H_3PO_4}$ (550 °C, Ar)	0.61	6.6	2.875	14.212
$NMC811_{H_2O}$ (650 °C, O_2)	0.61	4.9	2.875	14.214
$NMC811_{Al}$ (650 °C, O_2)	0.77	8.1	2.880	14.228
$NMC811_{H_3PO_4}$ (650 °C, O_2)	0.62	6.3	2.876	14.219

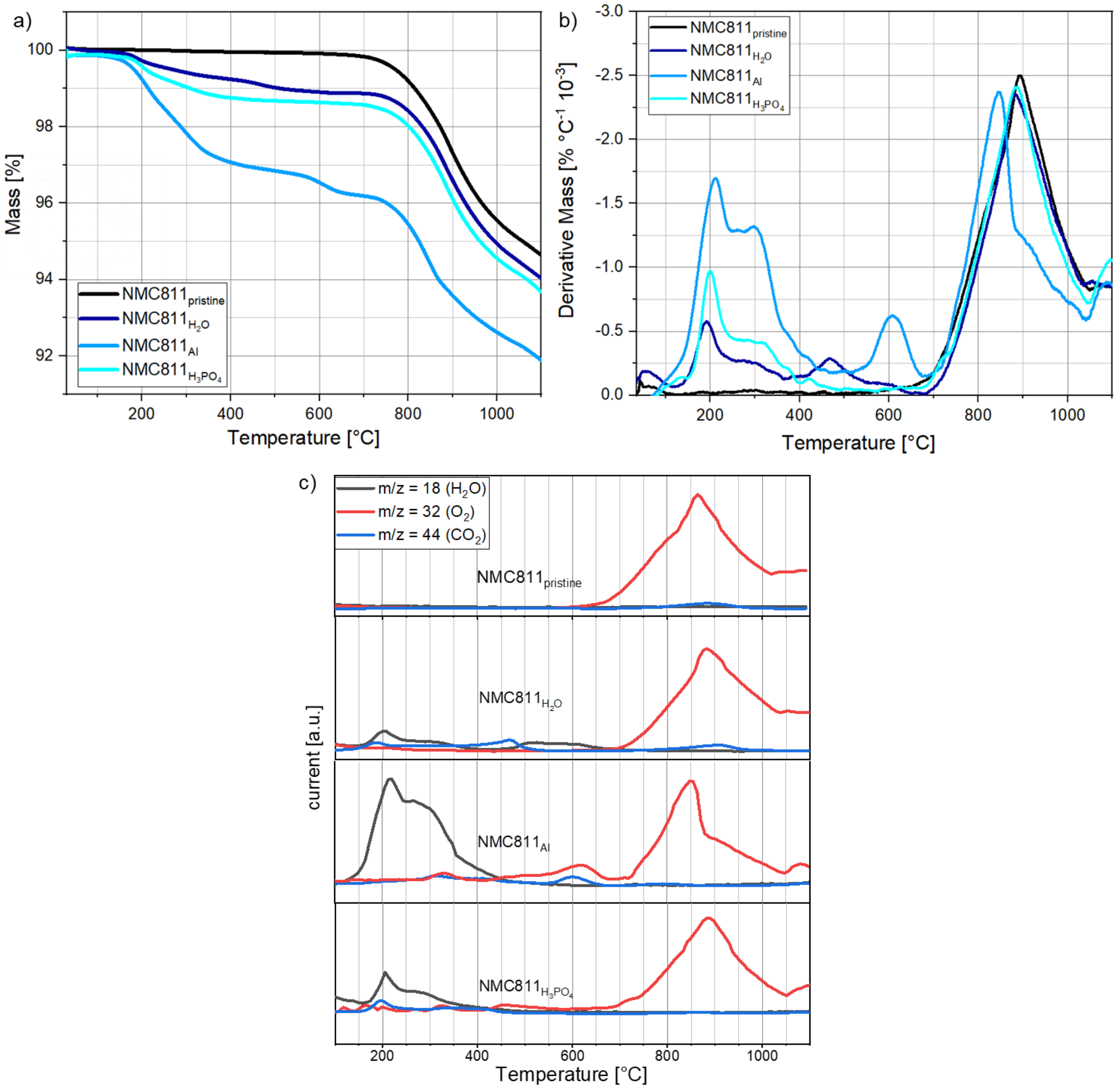

Figure S3: a) Mass loss curves from coupled TG-MS measurements (heating rate of 10 K/min), b) derivative of mass loss and c) mass signals. The m/z values of 18, 32 and 44 are attributed to H_2O, O_2 and CO_2, respectively.

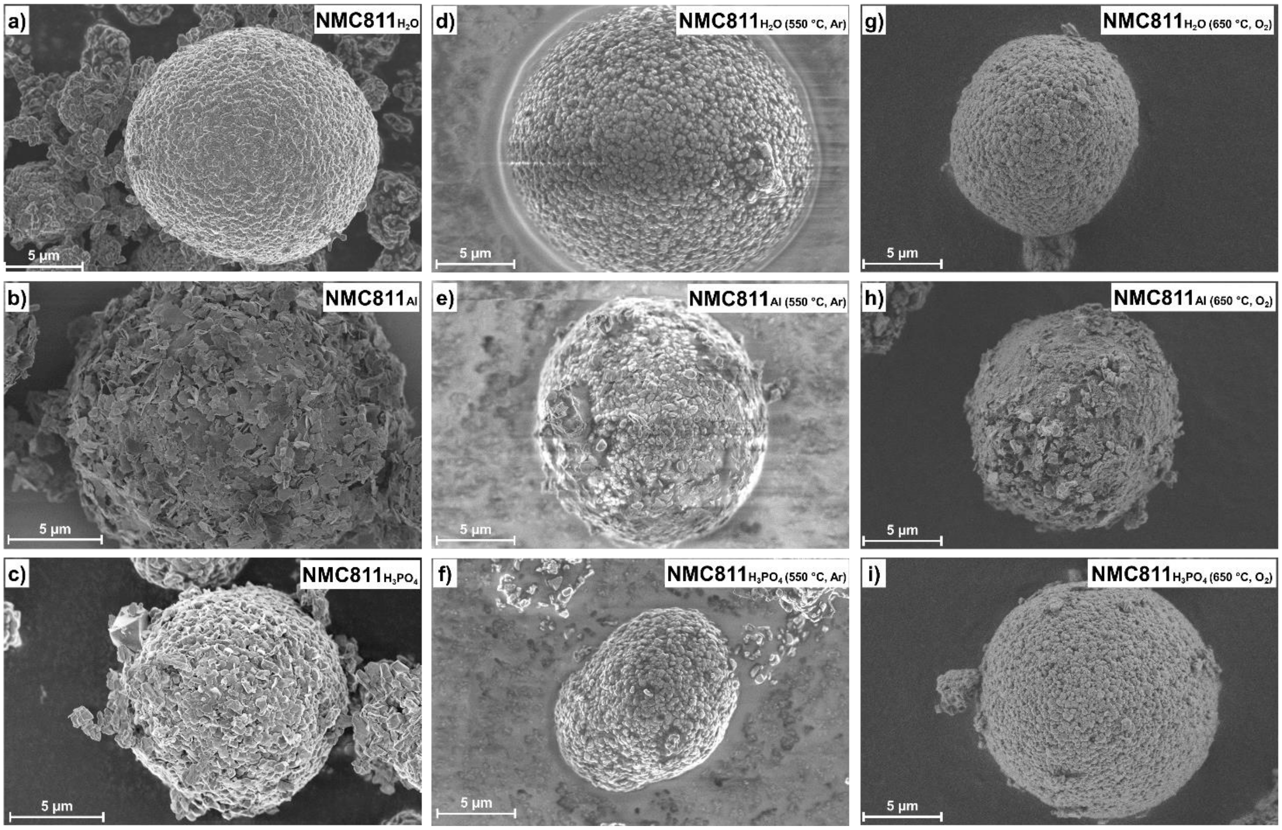

Figure S4: SEM images of the NMC811 from the different model recycling processes.

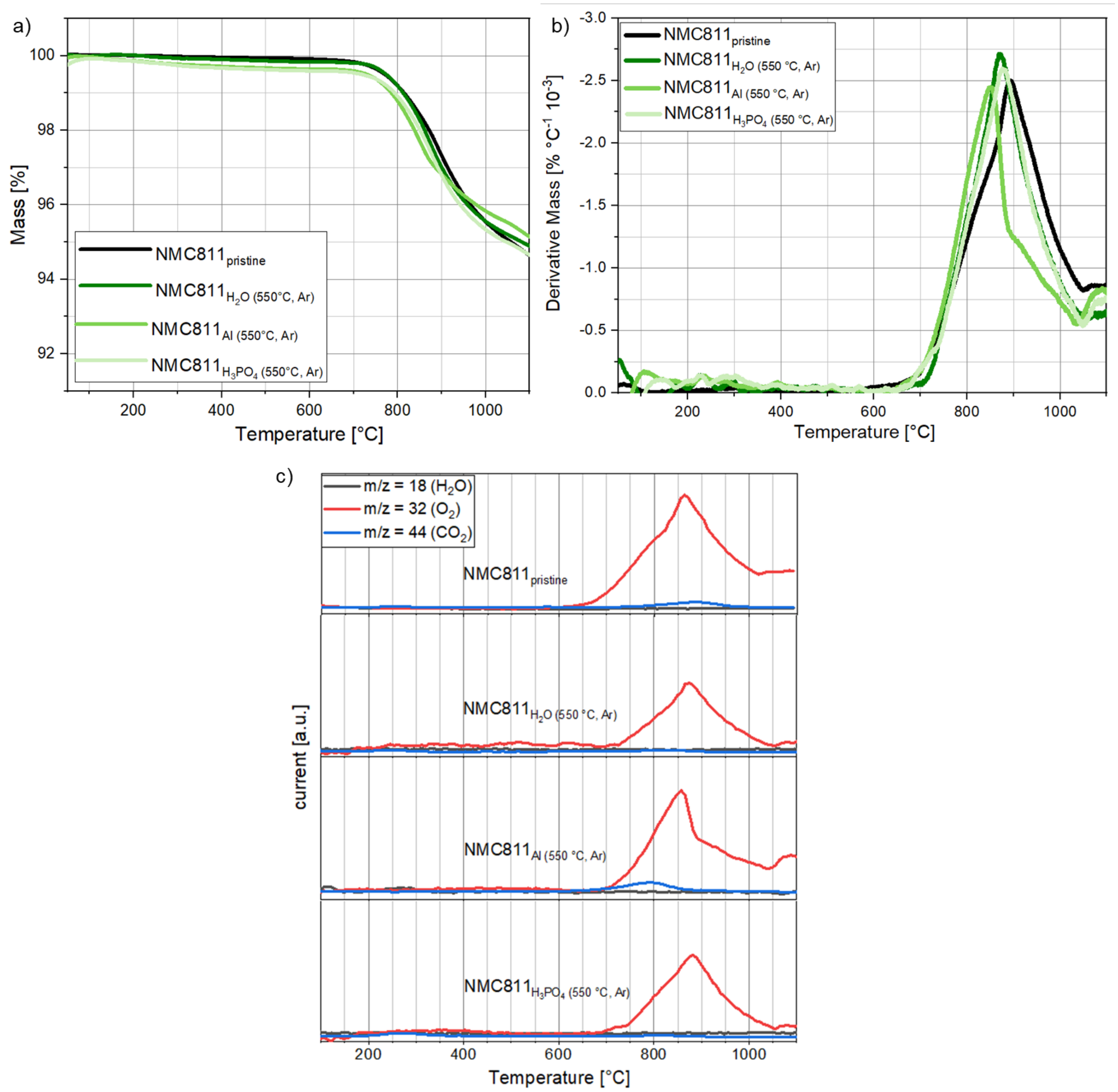

Figure S5: a) Mass loss curves from coupled TG-MS measurements (heating rate of 10 K/min), b) derivative of mass loss and c) mass signals. The m/z values of 18, 32 and 44 are attributed to H_2O, O_2 and CO_2, respectively.

Table 4: Values of fits for performed EIS measurements at 3.6 V.

	R_{IR}	R_{film}	R_{CT}
$NMC811_{pristine}$	1.5	20.0	140.1
$NMC811_{H_2O\ (550\ °C,\ Ar)}$	0.4	75.4	150.8
$NMC811_{Al\ (550\ °C,\ Ar)}$	0.3	12.3	372.4
$NMC811_{H_3PO_4\ (550\ °C,\ Ar)}$	1.1	56.9	200.2

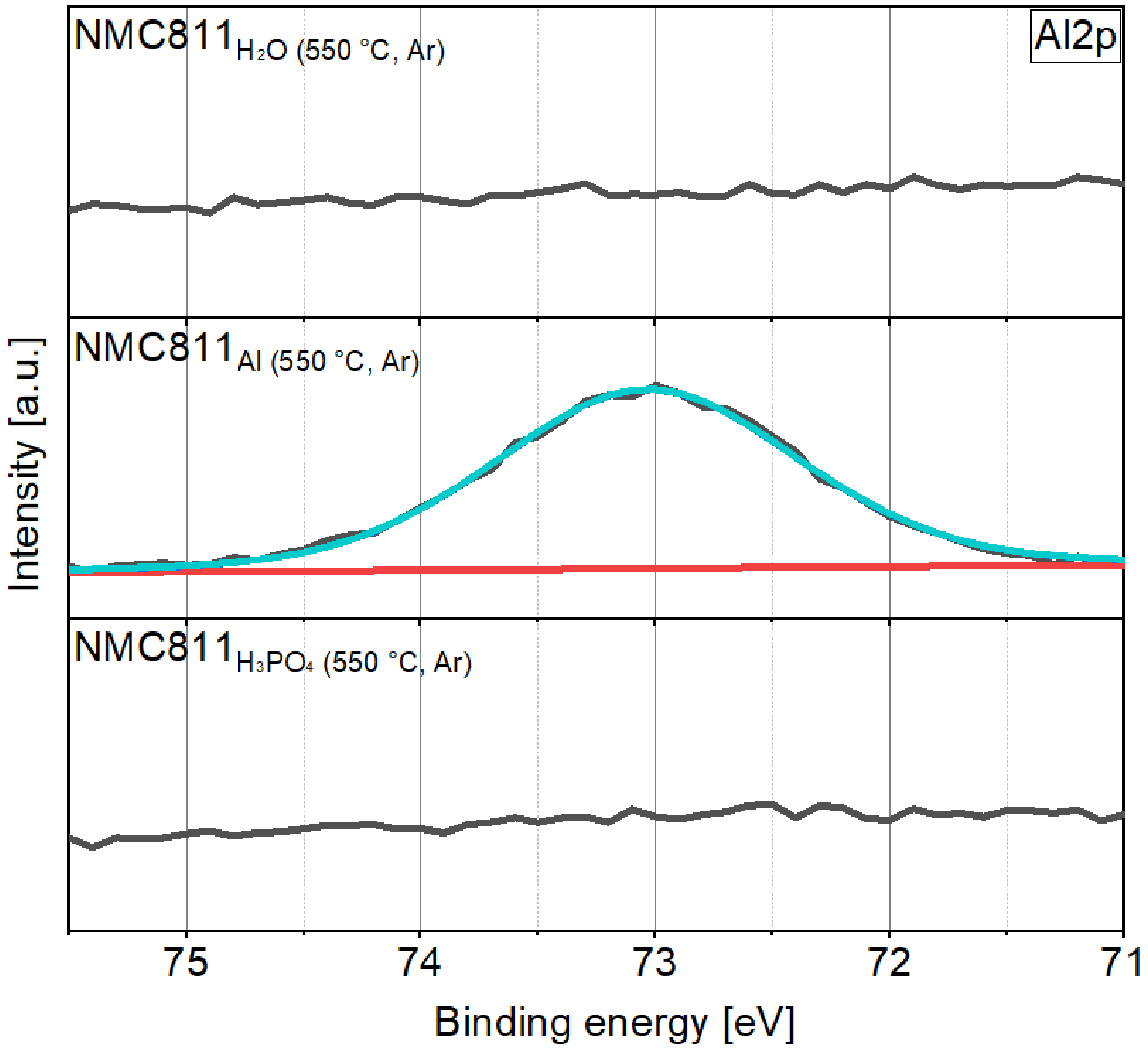

Figure S6: Al2p spectra of XPS measurement of heat treated NMC811 in argon atmosphere. 3

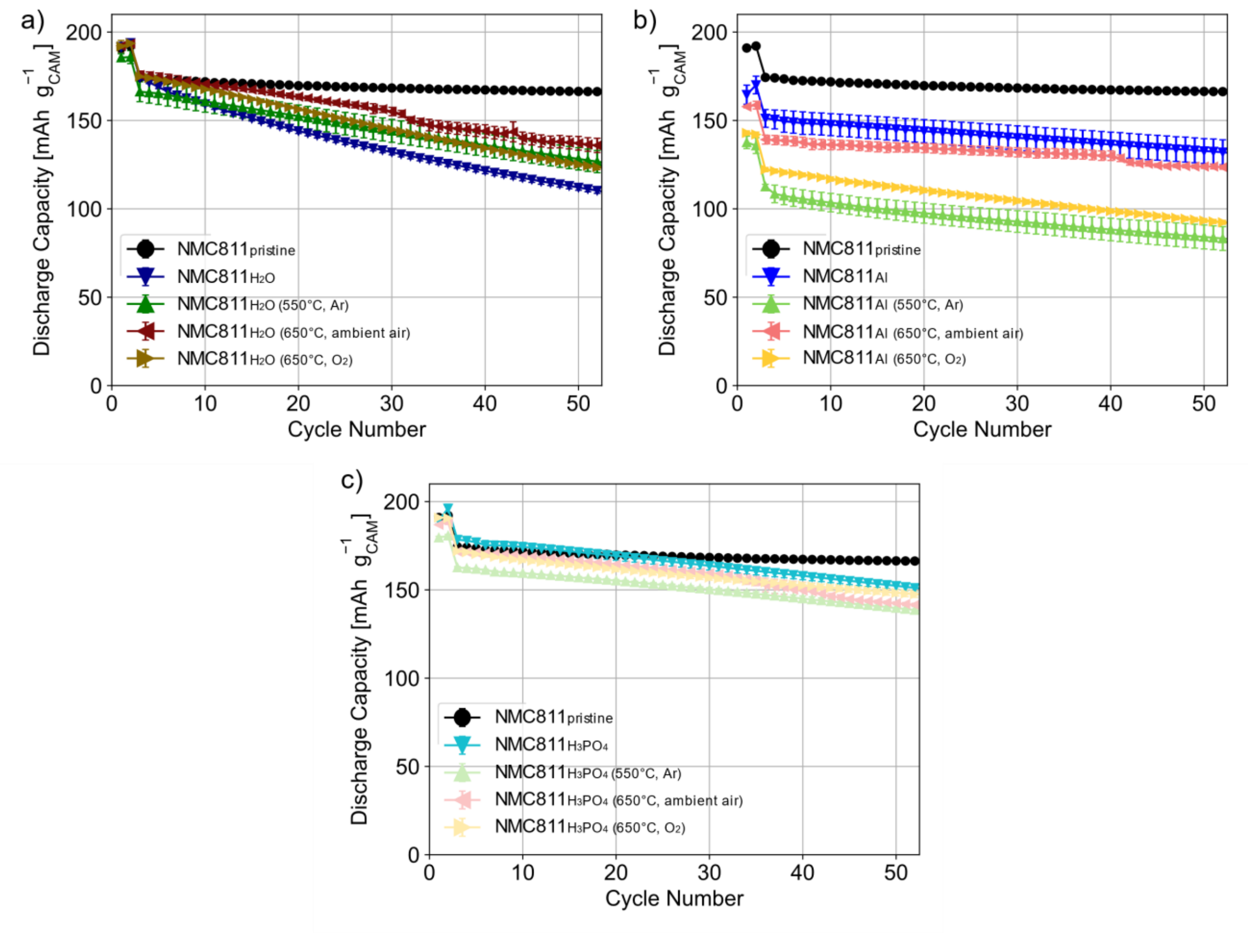

Figure S7: Electrochemical characterization of different heat treated NMC811 (550 °C in argon, 650 °C in ambient air, 650 °C in O_2). NMP/PVDF based cathodes were produced to "freeze" surface after processing. Further cathodes were tested in half cells against a lithium metal counter electrode to ensure the relithiation of the NMC811. Mean specific discharge capacity of three cells vs cycles in voltage window of 3.0-4.3 V at 25 °C. Error bars are the standard deviation of the three tested cells respectively. a) of all heat treated NMC811, which were in model system with only NMC811 and H_2O, b) of all heat treated NMC811, which were in model system with NMC811, H_2O and aluminum foil and c) of all heat treated NMC811, which were in model system with only NMC811, H_2O, aluminum foil and H_3PO_4.

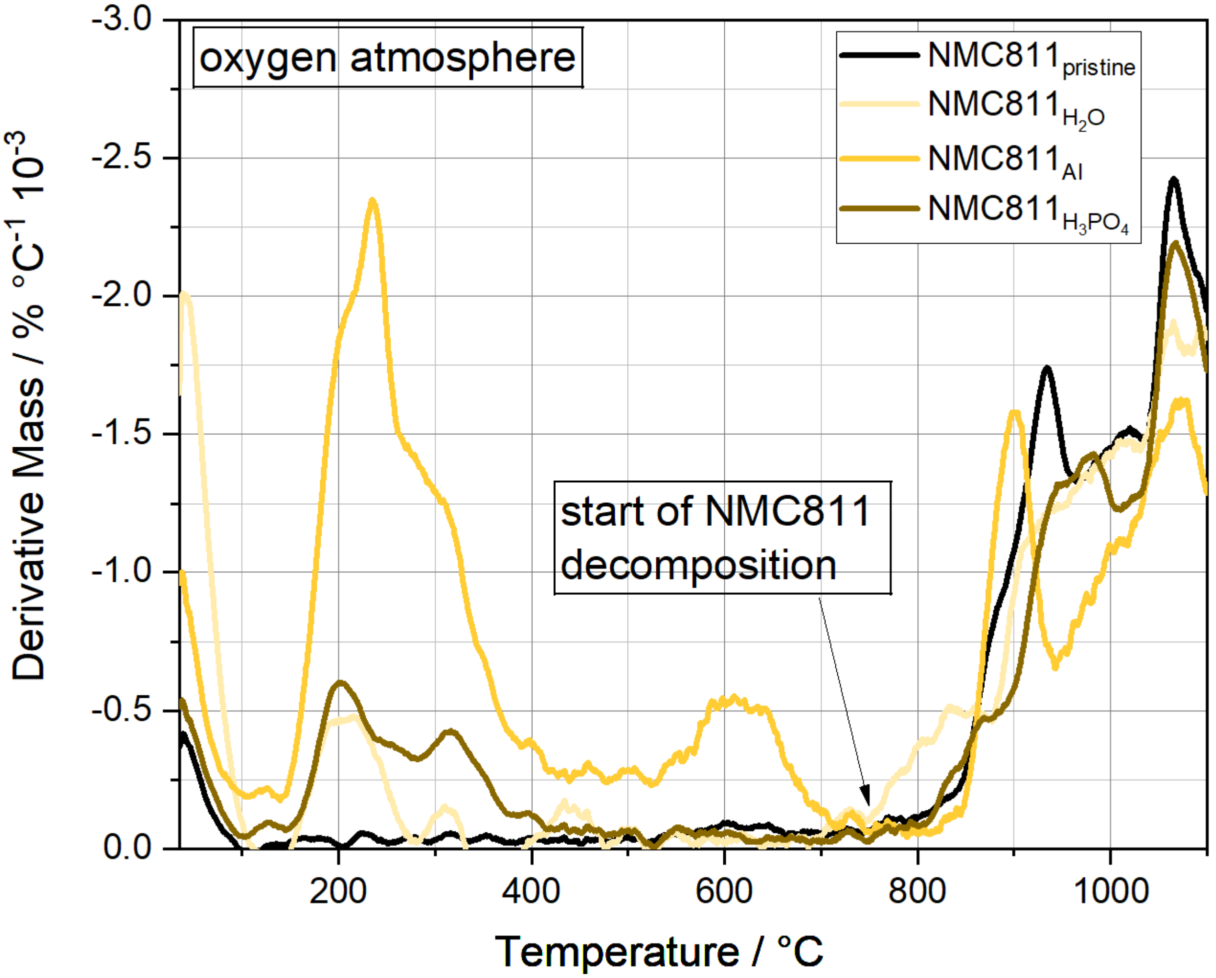

Figure S8: Derivative of mass loss of TG measurements (heating rate of 10 K/min) under oxygen atmosphere.
